# New 1,4-Dienonesteroids from the Octocoral *Dendronephthya* sp.

**DOI:** 10.3390/md17090530

**Published:** 2019-09-11

**Authors:** Thanh-Hao Huynh, Pei-Chin Chen, San-Nan Yang, Feng-Yu Lin, Tung-Pin Su, Lo-Yun Chen, Bo-Rong Peng, Chiung-Chin Hu, You-Ying Chen, Zhi-Hong Wen, Tung-Ying Wu, Ping-Jyun Sung

**Affiliations:** 1 Graduate Institute of Marine Biology, National Dong Hwa University, Pingtung 944, Taiwan; 2 National Museum of Marine Biology and Aquarium, Pingtung 944, Taiwan; 3 Doctoral Degree Program in Marine Biotechnology, National Sun Yat-sen University, Kaohsiung 804, Taiwan; 4 Department of Pediatrics, E-DA Hospital, School of Medicine, College of Medicine, I-SHOU University, Kaohsiung 804, Taiwan; 5 Department of Applied Chemistry, National Pingtung University, Pingtung 900, Taiwan; 6 Department of Chemistry, National Sun Yat-sen University, Kaohsiung 804, Taiwan; 7 Doctoral Degree Program in Marine Biotechnology, Academia Sinica, Taipei 115, Taiwan; 8 Department of Marine Biotechnology and Resources, National Sun Yat-sen University, Kaohsiung 804, Taiwan; 9 Chinese Medicine Research and Development Center, China Medical University Hospital, Taichung 404, Taiwan; 10 Graduate Institute of Natural Products, Kaohsiung Medical University, Kaohsiung 807, Taiwan

**Keywords:** *Dendronephthya*, dendronesterone, steroid, anti-inflammatory, iNOS

## Abstract

Two new steroids, dendronesterones D (**1**) and E (**2**), featuring with 1,4-dienone moiety, along with three known steroids, methyl 3-oxochola-4,22-diene-24-oate (**3**), 5α,8α-epidioxy-24(*S*)- methylcholesta-6,22-dien-3β-ol (**4**), and 5α,8α-epidioxy-24(*S*)-methylcholesta-6,9(11),22-trien-3β-ol (**5**), were isolated from an octocoral *Dendronephthya* sp. The structures of steroids **1** and **2** were elucidated by using spectroscopic methods and steroid **1** was found to exhibit significant in vitro anti-inflammatory activity in lipopolysaccharides (LPS)-induced RAW264.7 macrophage cells by inhibiting the expression of the iNOS protein.

## 1. Introduction

**Figure 1:**
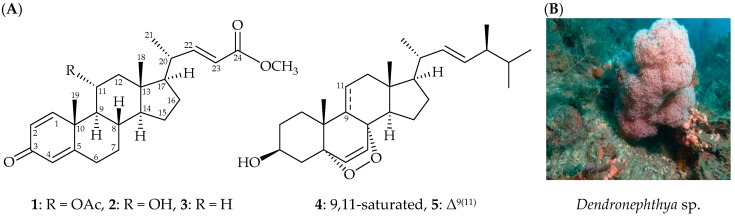
**(A**) Structures of dendronesterones D (**1**), E (**2**), methyl 3-oxochola-4,22-dien-24-oate (**3**), 5α,8α-epidioxy-24(*S*)-methylcholesta-6,22-dien-3β-ol (**4**), 5α,8α-epidioxy-24(*S*)-methylcholesta-6,9 (11),22-trien-3β-ol (**5**), and (**B**) A picture of octocoral *Dendronephthya* sp.

Marine invertebrates, particularly octocorals have been well recognized as a rich source of interesting steroid metabolites [1]. In continuation of research into new substances from marine invertebrates collected off the waters of Taiwan, a series of steroid derivatives have been isolated from the octocorals belonging to the genus *Dendronephthya* (phylum Cnidaria, class Anthozoa, order Alcyonacea, family Nephtheidae), octocorals distributed in the tropical and subtropical waters of the Indo-Pacific Ocean, and some of these metabolites were found to possess interesting bioactivities, such as cytotoxic [2] and anti-inflammatory activity [3,4]. Recently, chemical examination of an octocoral identified as *Dendronephthya* sp. resulted in the isolation of two new marine steroids, dendronesterones D (**1**) and E (**2**) (Figure 1), along with three known steroids, including an antifouling compound, methyl 3-oxochola-4,22-dien-24-oate (**3**), which was first isolated from a Japanese soft coral *Dendronephthya* sp. [5], and two cytotoxic metabolites, 5α,8α-epidioxy-24(*S*)-methylcholesta-6,22-dien-3β-ol (**4**) and 5α,8α-epidioxy-24(*S*)-methylcholesta- 6,9(11),22-trien-3β-ol (**5**) [6] (Figure 1), which were obtained from various marine invertebrates, such as sea squirts *Trididemnum inarmatum* [6] and *Ascidia nigra* [7], a hard coral *Dendrogyra cylindrus* [7], and a sponge *Thalysias juniperina* [7]. We reported herein the isolation and structural determination of steroids **1**–**5**. The ability of **1**–**5** to reduce the expression of the pro-inflammatory iNOS (inducible nitric oxide synthase) and COX-2 (cyclooxygenase-2) proteins in LPS (lipopolysaccharides)-stimulated RAW264.7 macrophage cells was determined.

## 2. Results

The new metabolite dendronesterone D (**1**) was isolated as a colorless oil, and its molecular formula was established as C_27_H_36_O_5_ (unsaturation degrees = 10) from a sodium adduct at *m/z* 463 in the (+)-ESIMS and further supported by the (+)-HRESIMS at *m/z* 463.24530 (calculated for C_27_H_36_O_5_ + Na, 463.24550). The ^13^C and DEPT spectroscopic data showed that this compound has 27 carbons (Table 1), including five methyls, five sp^3^ methylenes, six sp^3^ methines, two sp^3^ quaternary carbons, five sp^2^ methines, an sp^2^ quaternary carbon, two ester carbonyls, and a ketonic carbonyl. The IR spectrum revealed the presence of ester carbonyl (1724 cm^−1^) and α,β-unsaturated ketonic (1663 cm^−1^) groups. The ^1^H NMR spectra (Table 1) showed the presence of five olefinic methine protons (δ_H_ 6.78, d, *J* = 10.8 Hz; 6.74, dd, *J* = 15.6, 10.0 Hz; 6.13, dd, *J* = 10.8, 2.0 Hz; 6.10, dd, *J* = 2.0, 1.6 Hz; 5.79, d, *J* = 15.6 Hz) and an oxymethine proton (δ_H_ 5.17, ddd, *J* = 10.8, 10.8, 5.6 Hz). In addition, a carbonyl resonance at δ_C_ 169.7 further confirmed the existence of an ester group. The result of ^1^H NMR spectrum analysis indicated an acetate methyl (δ_H_ 2.01, 3H, s). The carbon signals at δ_C_ 156.2 (CH), 125.7 (CH), 186.2 (C), 124.6 (CH), and 167.1 (C) as well as the proton at δ_H_ 6.78 (1H, d, *J* = 10.8 Hz), 6.13 (1H, dd, *J* = 10.8, 2.0 Hz), and 6.10 (1H, dd, *J* = 2.0, 1.6 Hz) were characteristic signals of steroids with a 1,4-dien-3-one moiety in ring A [8,9,10,11,12].

**Figure 2:**
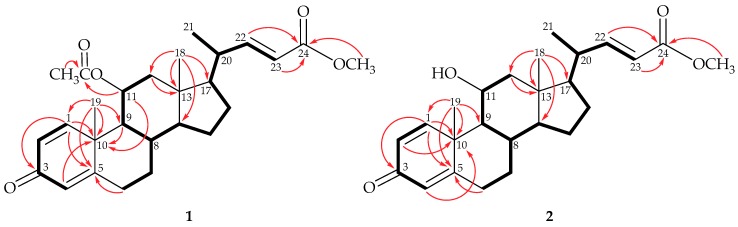
The COSY (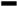) correlations and selective HMBC (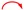) of steroids **1** and **2**.

^1^H NMR coupling information in the COSY spectrum of **1** enabled identification of H-1/H-2, H-2/H-4 (by a long range *W*-coupling), H_2_-6/H_2_-7/H-8/H-9/H-11/H_2_-12, H-8/H-14/H_2_-15/H_2_-16/H-17/ H-20/H-22/H-23, and H-20/H_3_-21 (Figure 2). These data, together with the key heteronuclear multiple bond correlation (HMBC) between protons and quaternary carbons, such as H-1/C-3; H-1, H_2_-6, H_3_-19/C-5; H-1, H-4, H-9, H-11, H_3_-19/C-10; H_2_-12, H_3_-18/C-13; and H-22, H-23/C-24, allowed us to establish the molecular skeleton of **1**. H-11 (δ_H_ 5.17) showed HMBC to C-10 and acetate carbonyl carbon at δ_C_ 169.7, demonstrating the acetoxy group at C-11. The methoxy group at C-24 was confirmed by the HMBC between the methyl protons of methoxy group (δ_H_ 3.72) and C-24 (δ_C_ 166.8).

**Figure 3:**
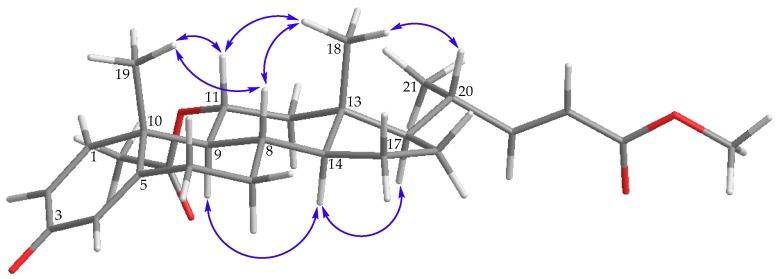
Selective protons with key NOESY correlations (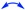) of **1**.

The relative configuration of **1** was elucidated by the NOE correlations observed in a NOESY experiment. H-8 showed correlations with both H_3_-18 and H_3_-19, and H_3_-18 exhibited correlations with H-11 and H-20; therefore, due to the β-orientation of Me-18 at C-13, all of H-8, H-11, H_3_-19, and H-20 should be positioned on the β-face. Furthermore, NOE responses between H-14 and H-9, and H-14 and H-17, were observed on the α-orientation of H-9, H-14, and H-17 (Figure 3) (Supplementary Materials, Figures S1–S10).

**Figure 4:**
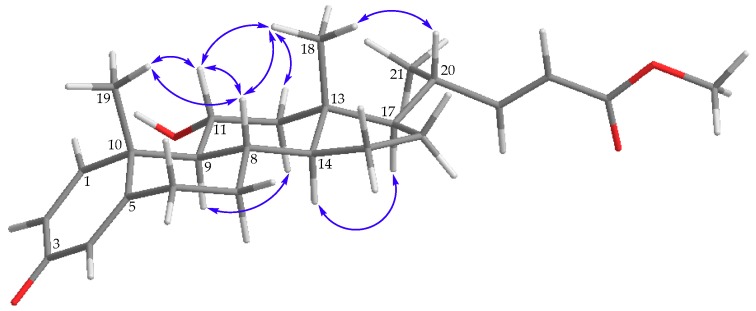
Selective protons with key NOESY correlations (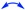) of **2**.

Steroid **2** (dendronesterone E) was isolated a colorless oil and was found to possess a molecular formula C_25_H_34_O_4_, as determined by (+)-HRESIMS at *m/z* 421.23502 (calculated for C_25_H_34_O_4_ + Na, 421.23493). IR absorptions at 3395, 1720, and 1657 cm^–1^ revealed the presence of hydroxy, ester, and α,β-unsaturated ketonic groups. Comparison of the ^1^H and ^13^C NMR data of **2** with those of **1** (Table 1) suggested that **2** is the 11-*O*-deacetyl analogue of **1**. This was further confirmed by the upfield shifts observed for H-11 (δ_H_ 3.99) and C-11 (δ_C_ 67.9) relative to those of **1** (δ_H_ 5.17; δ_C_ 69.8). The planar structure of **2**, including the positions of hydroxy group, carboxylate, and the olefinic double bonds, could be deduced from analysis of 2D NMR spectrum, including COSY and HMBC (Figure 2). The relative stereochemistry of **2** was established by the analysis of the NOE correlations in NOESY spectrum of **2**, as illustrated in Figure 4 (Supplementary Materials, Figures S11–S20).

The known steroids **3**–**5** were identified as methyl 3-oxochola-4,22-dien-24-oate [5], 5α,8α- epidioxy-24(*S*)-methylcholesta-6,22-dien-3β-ol [6,7], and 5α,8α-epidioxy-24(*S*)-methylcholesta-6,9 (11),22-trien-3β-ol [6,7], respectively, according to a comparison of their spectroscopic data analysis with the information described in the literature.

**Figure 5:**
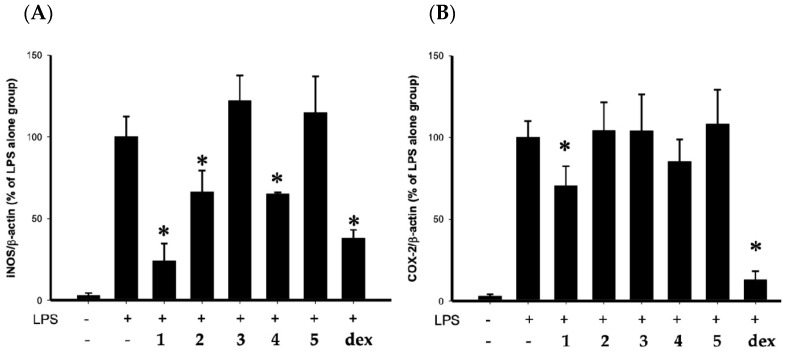
Effect of steroids **1**–**5** (10 μM) on pro-inflammatory (**A**) iNOS and (**B**) COX-2 protein expressions in the lipopolysaccharides (LPS)-stimulated murine macrophage cell line RAW264.7 by Western blotting analysis (Supplementary Materials, Figure S21). The relative intensity of iNOS/ COX-2 to β-actin bands was normalized to LPS-stimulated group, and cells treated with dexamethasone were used as a positive control. (* *p* < 0.05, significantly different from the LPS-stimulated group). Data are expressed as the mean ± SEM (*n* = 3 or 4).

Using an in vitro pro-inflammatory suppression assay, the effects of steroids **1**–**5** on the release of iNOS and COX-2 protein from LPS-stimulated RAW264.7 macrophage cells were assessed. The results of the in vitro pro-inflammatory suppression assay showed that steroid **1** at 10 μM suppressed the expression of iNOS/β-actin and COX-2/β-actin to 24.2 ± 10.6 and 70.4 ± 11.9%, as compared with LPS alone group (Figure 5). Compounds **1**–**5** did not significantly affect the viability of macrophage cells 16 h after treatments. 

## 3. Discussion

*Dendronephthya* spp. have been demonstrated to have a wide structural diversity of interesting steroids that possess various pharmacological properties, specifically in anti-inflammatory activities [13,14]. In our study of *Dendronephthya* sp., two previously unreported steroids, dendronesterones D (**1**) and E (**2**), were isolated together with the previously described marine steroids, methyl 3-oxochola-4,22-dien-24-oate (**3**), 5α,8α-epidioxy-24(*S*)-methylcholesta-6,22-dien-3β-ol (**4**), and 5α, 8α-epidioxy-24(*S*)-methylcholesta-6,9(11),22-trien-3β-ol (**5**). In the present study, the structures of new metabolites **1** and **2** were elucidated by spectroscopic methods and anti-inflammatory activities of steroids **1**–**5** were assessed using inhibition of pro-inflammatory iNOS and COX-2 release from macrophages. The results indicated that dendronesterone D (**1**) showed the most potent suppressive effects on iNOS release and steroids **2** and **3** showed more weak suppressive effects on iNOS/β-actin and COX-2/β-actin expression than those of **1**. The results suggested that the anti- inflammatory activities of steroids **1**–**3** were mainly reliant on the functional group at C-11. Furthermore, steroid **5** was found to be inactive in terms of reducing the expression of iNOS/β-actin, indicating that the anti-inflammatory activities of steroids **4** and **5** are dependent on the existence of the carbon–carbon double bond between C-9/11. 

## 4. Experimental Section

### 4.1. General Experimental Procedures

Optical rotations were measured on a Jasco P-1010 digital polarimeter (Japan Spectroscopic Corporation, Tokyo, Japan); infrared spectra were recorded on a Thermo, Nicolet iS5 FT-IR (Thermo Scientific Nicolet, Waltham, MA, USA); peaks are reported in cm^–1^. The NMR spectra were recorded on a Jeol FT-NMR (model ECZ400S, Tokyo, Japan) spectrometer operating at 400 MHz for ^1^H and 100 MHz for ^13^C, using the residual CHCl_3_ signal (δ_H_ 7.26 ppm) as an internal standard for ^1^H NMR and CDCl_3_ (δ_C_ 77.1 ppm) for ^13^C NMR; coupling constants (*J*) are given in Hz. ESIMS and HRESIMS were recorded using a Bruker 7 Tesla solariX FTMS system (Bremen, Germany). Column chromatography was performed on silica gel (230–400 mesh, Merck). TLC was carried out on precoated Kieselgel 60 F_254_ (0.25 mm, Merck); spots were visualized by spraying with 10% H_2_SO_4_ solution followed by heating. Normal-phase HPLC (NP-HPLC) was performed using a system comprised of a Hitachi 5110 pump (Hitachi, Tokyo, Japan) and a Rheodyne 7725 injection port (Rheodyne, Rohnert Park, CA, USA). A normal-phase column (Luna, 5 μm, Silica (2) 100Å, 250 × 10 mm) was used for NP-HPLC. Reversed-phase HPLC (RP-HPLC) was performed using a system comprised of a Hitachi L-2130 pump, a Hitachi L-2455 photodiode array detector, and a Rheodyne 7725 injection port. A reverse phase column (Luna, 5 μm C18(2) 100Å, 250 × 21.2 mm) was used for RP-HPLC.

### 4.2. Animal Material

Specimens of the octocoral *Dendronephthya* sp. were collected by hand using self-contained underwater breathing apparatus (SCUBA) diving off the northeast coast of Taiwan in August 30th, 2018, and stored in a –20 °C freezer until extraction. A voucher specimen (NMMBA-TW-SC-2018-018) was deposited in the National Museum of Marine Biology and Aquarium (NMMBA), Taiwan. This organism was identified by comparison with previous descriptions [15]. 

### 4.3. Extraction and Separation

Sliced bodies of *Dendronephthya* sp. (wet weight 748.7 g; dry weight 186.8 g) were extracted with a 1:1 mixture of methanol (MeOH) and dichloromethane to give 12.2 g of crude extract which was partitioned between ethyl acetate (EtOAc) and H_2_O. The EtOAc extract (2.4 g) was applied on silica gel column chromatography and eluted with gradients of *n*-hexane/EtOAc (100:1—pure EtOAc, stepwise), to furnish 14 fractions (fractions: A–N). Fractions I, L, and M were purified by NP-HPLC using a mixture of *n*-hexane/acetone, 4:1 for fractions I and M, and 6:1 for fraction L, to afford **3** (8.5 mg), **1** (6.6 mg), and **2** (3.0 mg), respectively. Fraction J was purified by NP-HPLC using a mixture of *n*-hexane/acetone (5:1) to yield nine fractions J1–J9. Fraction J6 was separated by RP-HPLC using a mixture of MeOH/H_2_O (95:5) to afford **5** (0.6 mg) and **4** (1.5 mg), respectively. 

Dendronesterone D (**1**): Colorless oil: ${\left[\alpha \right]}_{D}^{25}$ +79 (*c* 0.3, CHCl_3_); IR (ATR) ν_max_ 1724, 1663 cm^−^^1^; ^1^H (400 MHz, CDCl_3_) and ^13^C (100 MHz, CDCl_3_) NMR data, see Table 1; ESIMS *m/z* 463 [M + Na]^+^; HRESIMS *m/z* 463.24530 (calculated for C_27_H_3__6_O_5_ + Na, 463.24550).

Dendronesterone E (**2**): Colorless oil: ${\left[\alpha \right]}_{D}^{25}$ +57 (*c* 0.08, CHCl_3_); IR (ATR) ν_max_ 3395, 1720, 1657 cm^−^^1^; ^1^H (400 MHz, CDCl_3_) and ^13^C (100 MHz, CDCl_3_) NMR data, see Table 1; ESIMS *m/z* 421 [M + Na]^+^; HRESIMS *m/z* 421.23502 (calculated for C_25_H_34_O_4_ + Na, 421.23493).

Methyl 3-oxochola-4,22-dien-24-oate (**3**): Colorless oil: ${\left[\alpha \right]}_{D}^{25}$ +52 (*c* 0.2, CHCl_3_) (ref. [5], ${\left[\alpha \right]}_{D}^{22}$ +53.6 (*c* 0.28, CHCl_3_)); IR (ATR) ν_max_ 1721, 1662 cm^−^^1^; ^1^H (400 MHz, CDCl_3_) and ^13^C (100 MHz, CDCl_3_) NMR data were found to be in full agreement with those reported previously [5]; ESIMS *m*/*z* 405 [M + Na]^+^.

5α,8α-Epidioxy-24(*S*)-methylcholesta-6,22-dien-3β-ol (**4**): Amorphous powder: ${\left[\alpha \right]}_{D}^{24}$ −6 (*c* 0.07, CHCl_3_); IR (ATR) ν_max_ 3375 cm^−^^1^; ^1^H (400 MHz, CDCl_3_) and ^13^C (100 MHz, CDCl_3_) NMR data were found to be in full agreement with those reported previously [7]; ESIMS *m*/*z* 451 [M + Na]^+^.

5α,8α-Epidioxy-24(*S*)-methylcholesta-6,9(11)22-trien-3β-ol (**5**): Amorphous powder: ${\left[\alpha \right]}_{D}^{24}$ +214 (*c* 0.2, CHCl_3_); IR (ATR) ν_max_ 3391 cm^−^^1^; ^1^H (400 MHz, CDCl_3_) and ^13^C (100 MHz, CDCl_3_) NMR data were found to be in full agreement with those reported previously [7]; ESIMS *m*/*z* 449 [M + Na]^+^.

### 4.4. In Vitro Anti-Inflammatory Assay

The anti-inflammatory activity method used was modified from our previous studies [16,17,18]. We examined the effects of steroids **1**–**5** on pro-inflammatory iNOS and COX-2 protein expressions in LPS-stimulated RAW264.7 cells by Western blotting analysis. RAW264.7 were obtained from the American Type Culture Collection (ATCC TIB-71, Mannassas, VA, USA). The cells was seeded in 10-cm dishes at a density of 1 × 10^6^ cells. The inflammatory response was induced by incubation of LPS (0.01 μg/mL) for 16h. For the anti-inflammatory activity assay, steroids **1**–**5** and dexamethasone (as positive control) at 10 μM were added to the cells 10 min before LPS challenge. After 16 h, the cells were then washed with ice-cold phosphate-buffered saline, lysed in lysis buffer (50 mM Tris, pH 7.5, 150 mM NaCl, 1% Triton X-100, 100 μg/mL phenylmethylsulfonyl fluoride and 1 μg/mL aprotinin), and centrifuged at 20,000 g for 30 min at 4 °C. The supernatants were reserved for western blotting. Protein concentrations were measured by the DC protein assay kit (Bio-Rad, Hercules, CA, USA). An equal volume of sample buffer (2% 2-mercaptoethanol, 2% sodium dodecyl suflate (SDS), 0.1% bromophenol blue, 10% glycerol, and 50 mM Tris-HCl (pH 7.2)) was added to the samples, and the protein lysates (50 μg) loaded onto tricine SDS-polyacrylamide (7% or 10%) gel. After electrophoresis, proteins were transferred to polyvinylidene difluoride (PVDF) membranes (Immobilon-P; pore size, 0.45 μM; Millipore, Bedford, MA, USA) at 135 mA overnight at 4 °C in transfer buffer (50 mM Tris-HCl, 380 mM glycine, 1% SDS, 20% methanol). The PVDF was incubated overnight at 4 °C with the anti-iNOS, anti-COX-2, or anti-β-actin antibodies. A horseradish peroxidase-conjugated secondary antibody was used for detection. Anti-iNOS (catalog no. 160862) and anti-COX-2 (catalog no. 160106) antibodies were purchased from Cayman Chemical Company (Ann Arbor, MI, USA). The β-actin antibody (catalog no. Actin sigma A5441) was purchased from Sigma-Aldrich (St. Louis, MO, USA). Immunoreactive bands were visualized by enhanced chemiluminescence (ECL kit; Millipore) and the BioChemi Imaging System and relative densitometric quantification was performed using LabWorks v6.2 (UVP, Upland, CA, USA). Bands for iNOS, COX-2, and β-actin antibodies were recognized at ~135, ~72, and ~45 kDa, respectively. The experiment was repeated 3−4 times and data presented as the mean ± standard error of the mean (SEM). For statistical analysis of immunoblot, the integrated optical density of the LPS group was set to 100%, and β-actin was used to verify that equivalent amounts of protein were loaded in each lane. The data was analyzed by analysis of variance (ANOVA) with the Student–Newman– Keuls post hoc test for multiple comparisons. The difference was significant when *p* was less than 0.05.

### 4.5. Cell Viability

The RAW264.7 macrophage cell viability was determined after treatment with alamar blue (invitrogen, Carlsbad, CA, USA) [18], a tetrazolium dye that is reduced by living cells to fluorescent products. This assay is similar in principle to the cell viability assay using 3-(4,5-dimethyldiazol-2- yl)-2,5-diphenyltetrazolium bromide and has been validated as an accurate measure of the survival of RAW264.7 macrophage cells. 

## Figures and Tables

**Table 1:** ^1^H (400 MHz, CDCl_3_) and ^13^C (100 MHz, CDCl_3_) NMR data for steroids **1** and **2**.

	1			2	
C/H	δ_H_ (*J* in Hz)	δ_C_, Type		δ_H_ (*J* in Hz)	δ_C_, Type
1	6.78 d (10.8)	156.2 (CH)		7.74 d (10.8)	158.8 (CH)
2	6.13 dd (10.8, 2.0)	125.7 (CH)		6.15 dd (10.8, 2.0)	125.1 (CH)
3		186.2 (C)			183.8 (C)
4	6.10 dd (2.0, 1.6)	124.6 (CH)		6.09 dd (2.0, 1.2)	124.6 (CH)
5		167.1 (C)			167.9 (C)
6α β	2.38 ddd (13.2, 4.4, 2.4) 2.48 ddd (13.2, 13.2, 4.8, 0.8)	32.8 (CH_2_)		2.36 ddd (13.2, 4.4, 2.8) 2.45 ddd (13.2, 13.2, 5.2, 1.6)	33.2 (CH_2_)
7α/β	1.14 m; 1.97 m	33.3 (CH_2_)		1.09 m; 1.96 m	33.4 (CH_2_)
8	1.72 m	34.4 (CH)		1.61 m	34.3 (CH)
9	1.39 dd (10.8, 10.8)	56.3 (CH)		1.09 dd (10.4, 10.4)	60.2 (CH)
10		43.4 (C)			44.0 (C)
11	5.17 ddd (10.8, 10.8, 5.6)	69.8 (CH)		3.99 m	67.9 (CH)
12α/β	1.00 dd (12.4, 10.8); 2.13 dd (12.4, 5.6)	44.7 (CH_2_)		1.00 m; 2.10 dd (12.0, 4.8)	50.0 (CH_2_)
13		42.5 (C)			42.9 (C)
14	1.14 m	53.9 (CH)		1.09 m	54.5 (CH)
15α/β	1.67 m; 1.16 m	23.9 (CH_2_)		1.63 m; 1.18 m	24.0 (CH_2_)
16α/β	1.92 m; 1.36 m	27.4 (CH_2_)		1.93 m; 1.38 m	27.7 (CH_2_)
17	1.30 dd (9.2, 9.2)	55.3 (CH)		1.32 m	55.3 (CH)
18	0.76 s	12.9 (CH_3_)		0.73 s	13.3 (CH_3_)
19	1.26 s	18.7 (CH_3_)		1.25 s	18.7 (CH_3_)
20	2.24 m	39.5 (CH)		2.25 m	40.0 (CH)
21	0.97 d (6.4)	19.8 (CH_3_)		0.99 d (6.4)	20.0 (CH_3_)
22	6.74 dd (15.6, 10.0)	154.0 (CH)		6.84 dd (15.6, 10.4)	154.8 (CH)
23	5.79 d (15.6)	119.3 (CH)		5.81 d (15.6)	119.2 (CH)
24		166.8 (C)			167.2 (C)
OAc-11	2.01 s	169.7 (C) 21.6 (CH_3_)			
OMe-24	3.72 s	51.3 (CH_3_)		3.74 s	51.5 (CH_3_)

## Supplementary Materials

The Supplementary Materials are available online at https://www.mdpi.com/1660-3397/17/9/530/s1, Figure S1: ESIMS spectrum of compound **1**, Figure S2: HRESIMS spectrum of compound **1**, Figure S3: IR spectrum of compound **1**, Figure S4: ^1^H NMR spectrum (400 MHz) of compound **1** in CDCl_3_, Figure S5: ^13^C NMR spectrum (100 MHz) of compound **1** in CDCl_3_, Figure S6: DEPT spectrum of compound **1** in CDCl_3_, Figure S7: HSQC spectrum of compound **1** in CDCl_3_, Figure S8: HMBC spectrum of compound **1** in CDCl_3_, Figure S9: ^1^H–^1^H COSY spectrum of compound **1** in CDCl_3_, Figure S10: NOESY spectrum of compound **1** in CDCl_3_, Figure S11: ESIMS spectrum of compound **2**, Figure S12: HRESIMS spectrum of compound **2**, Figure S13: IR spectrum of compound **2**, Figure S14: ^1^H NMR spectrum (400 MHz) of compound **2** in CDCl_3_, Figure S15: ^13^C NMR spectrum (100 MHz) of compound **2** in CDCl_3_, Figure S16: DEPT spectrum of compound **2** in CDCl_3_, Figure S17: HSQC spectrum of compound **2** in CDCl_3_, Figure S18: HMBC spectrum of compound **2** in CDCl_3_, Figure S19: ^1^H–^1^H COSY spectrum of compound **2** in CDCl_3_, Figure S20: NOESY spectrum of compound **2** in CDCl_3_, Figure S21: The raw Western blotting data (pictures) of steroids **1**–**5**.
