# Supplementary material for: New 1,4-Dienonesteroids from the Octocoral Dendronephthya sp
Source: Mar Drugs. 2019 Sep 11;17(9):530. doi: 10.3390/md17090530 (PMC6780379; doi:10.3390/md17090530)
Supplement: Supplementary file 1 [file marinedrugs-17-00530-s001.pdf]

Supplementary materials

S1. ESIMS spectrum of compound **1**……………………………………………….2

S2. HRESIMS spectrum of compound **1** 3

S3. IR spectrum of compound **1** 4

S4. ^{1}H NMR spectrum (400 MHz) of compound **1** in $CDCl_3$ 5

S5. ^{13}C NMR spectrum (100 MHz) of compound **1** in $CDCl_3$......................................6

S6. DEPT spectrum (100 MHz) of compound **1** in $CDCl_3$...7

S7. HSQC spectrum of compound **1** in $CDCl_3$……………………………………..8

S8. HMBC spectrum of compound **1** in $CDCl_3$……………………………………….9

S9. ^{1}H-^{1}H COSY spectrum of compound **1** in $CDCl_3$………………………………..10

S10. NOESY spectrum of compound **1** in $CDCl_3$……………………………………11

S11. ESIMS spectrum of compound **2**..12

S12. HRESIMS spectrum of compound **2**..13

S13. IR spectrum of compound **2** 14

S14. ^{1}H NMR spectrum (400 MHz) of compound **2** in $CDCl_3$...................................15

S15. ^{13}C NMR spectrum (100 MHz) of compound **2** in $CDCl_3$...................................16

S16. DEPT spectrum (100 MHz) of compound **2** in $CDCl_3$.......................................17

S17. HSQC spectrum of compound **2** in $CDCl_3$...18

S18. HMBC spectrum of compound **2** in $CDCl_3$..19

S19. ^{1}H-^{1}H COSY spectrum of compound **2** in $CDCl_3$ 20

S20. NOESY spectrum of compound **2** in $CDCl_3$ 21

S21. The raw Western blotting data (pictures) of steroids **1-5**....................................22

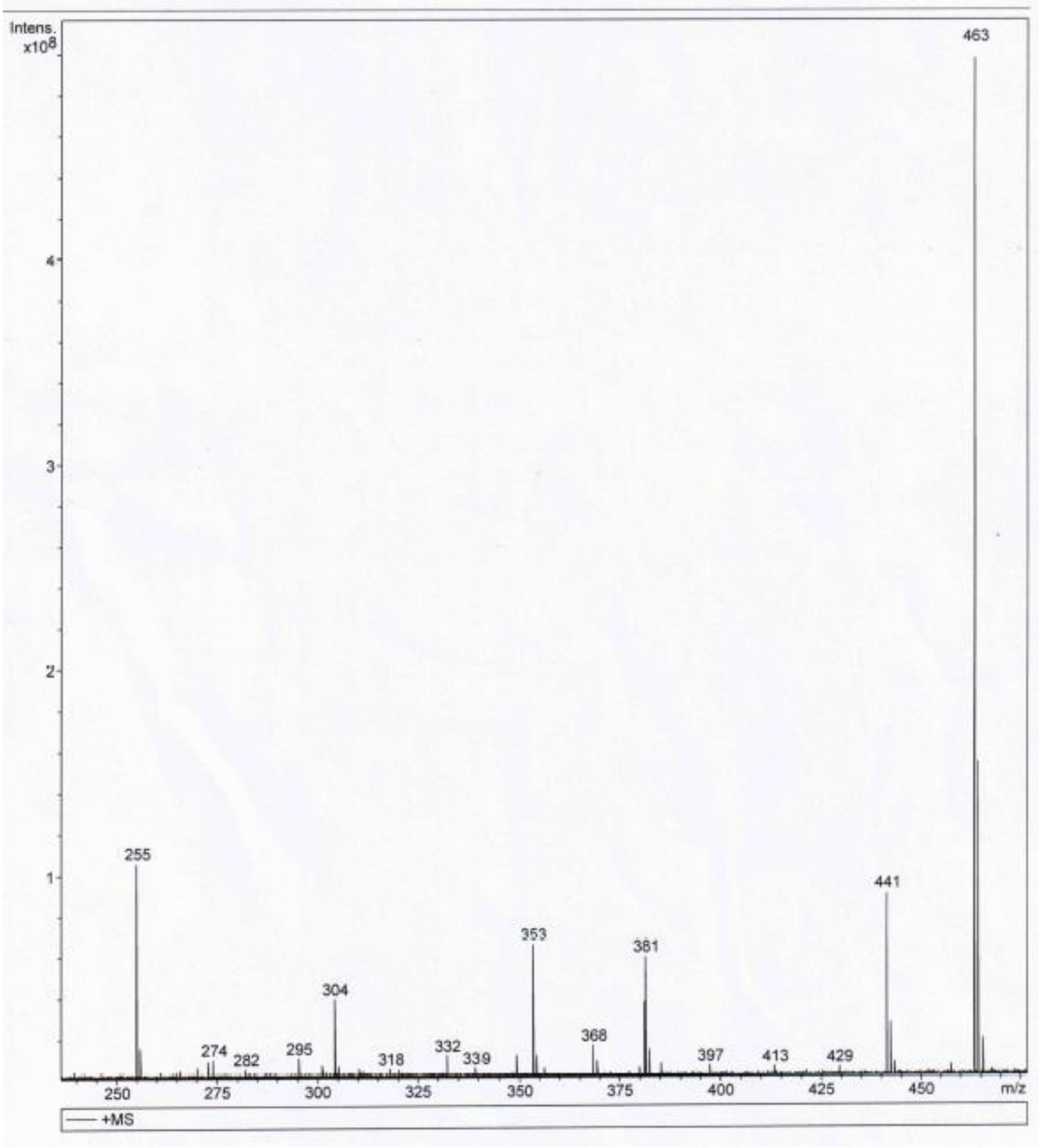

S1. ESIMS spectrum of compound **1**

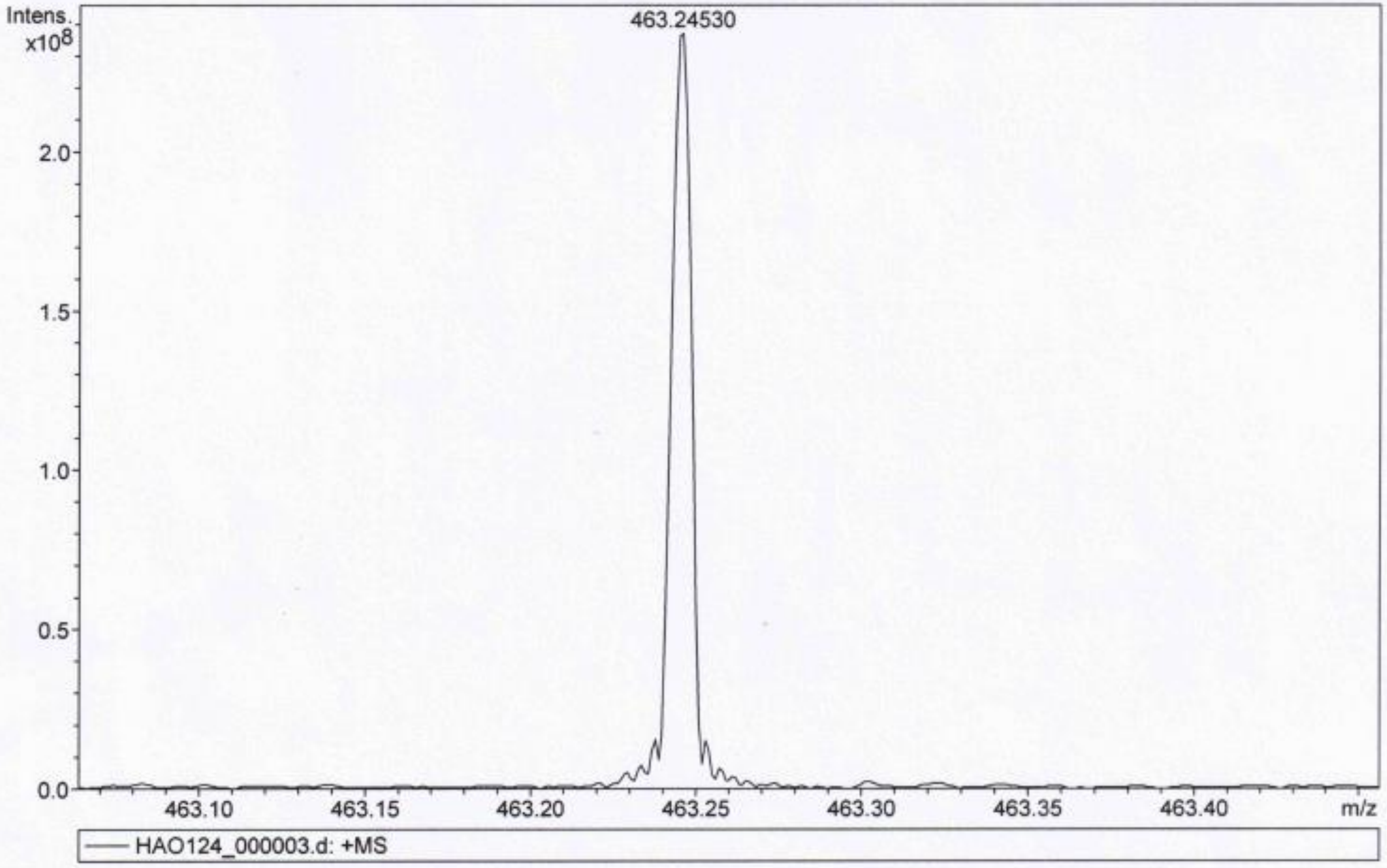

Meas. m/z	#	Formula	Score	m/z	err [mDa]	err [ppm]	mSigma	rdb	e⁻ Conf	N-Rule
463.24530	1	C 27 H 36 Na O 5	100.00	463.24550	0.20	0.43	3.4	9.5	even	ok

S2. HRESIMS spectrum of compound **1**

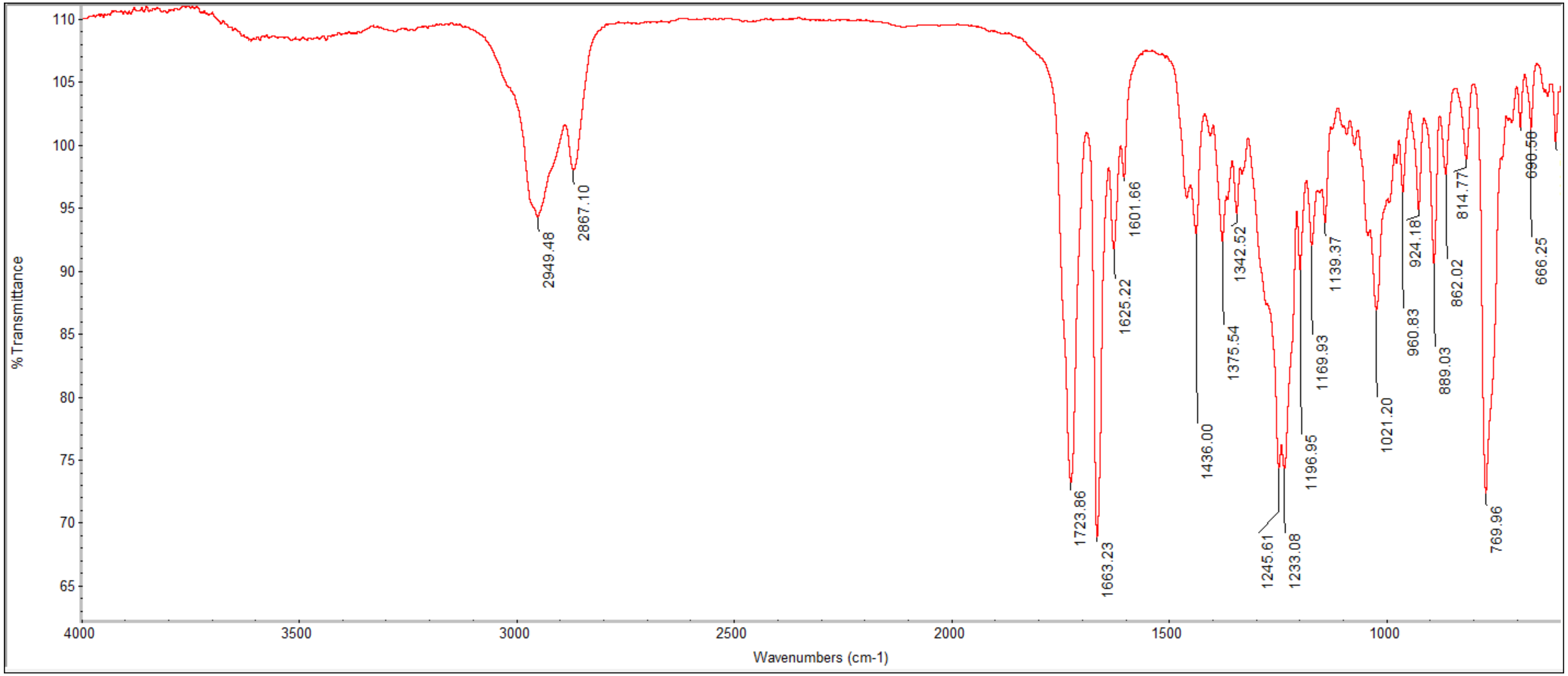

S3. IR spectrum of compound **1**

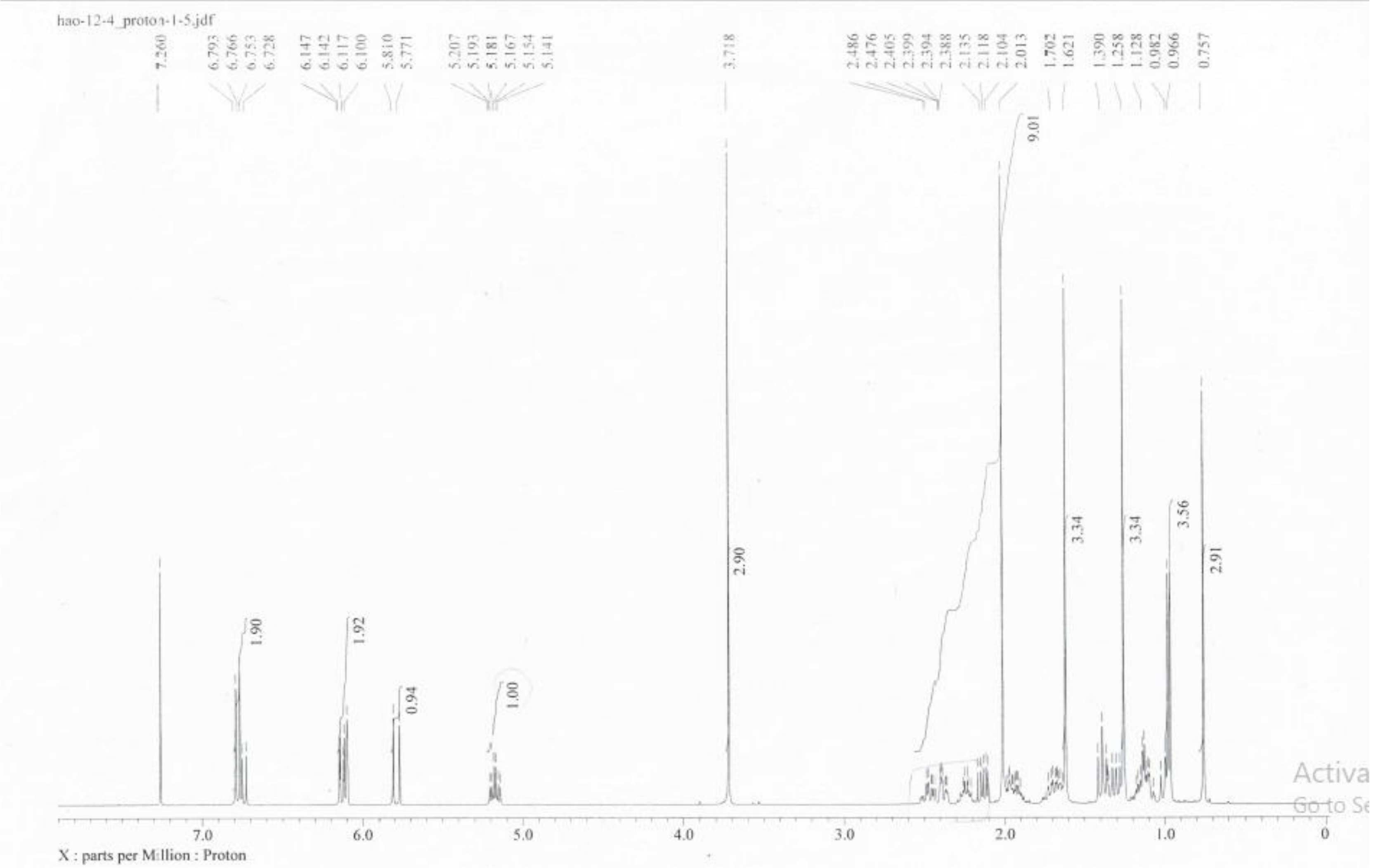

S4. ^{1}H NMR spectrum (400 MHz) of compound **1** in $CDCl_3$

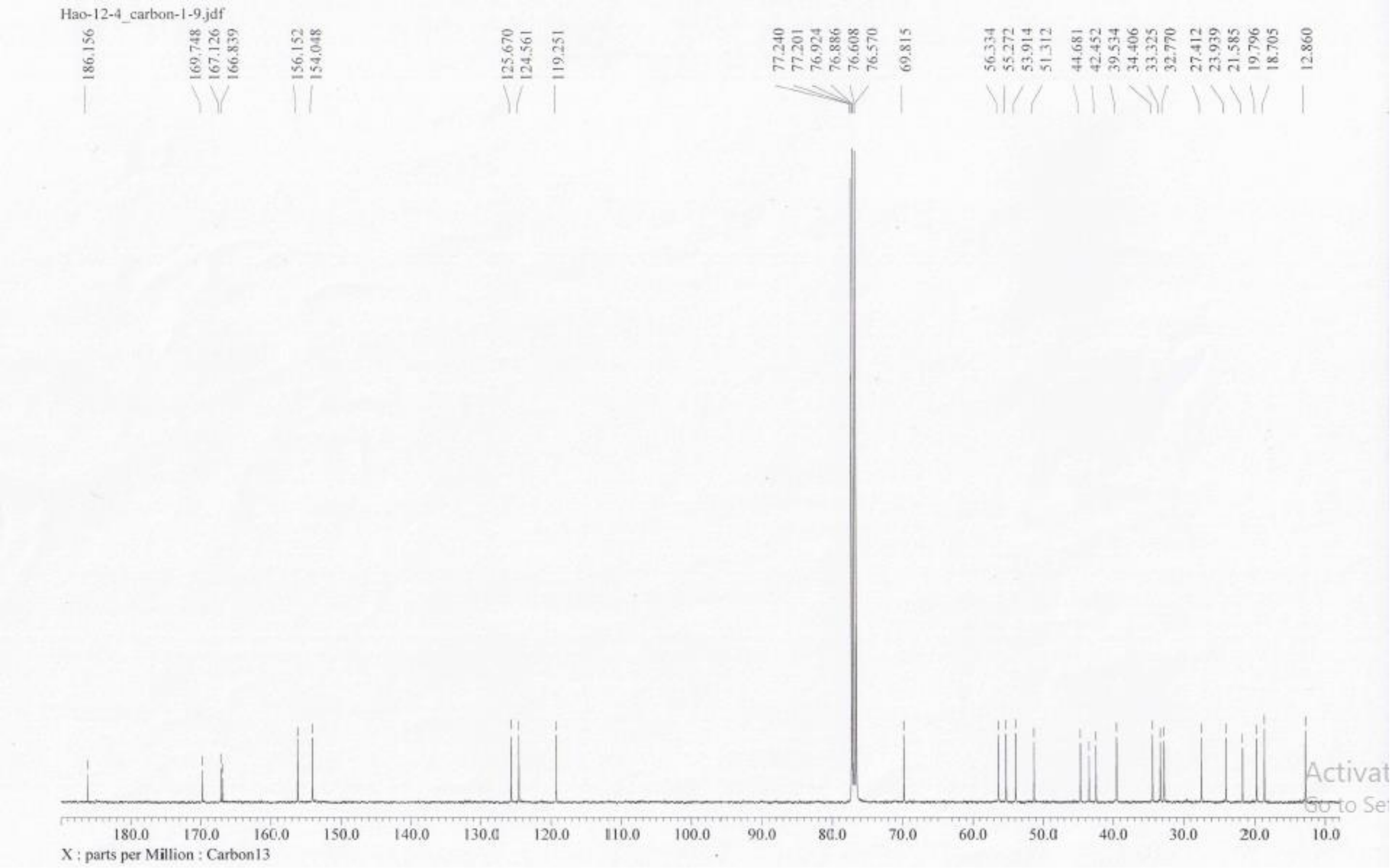

S5. ^{13}C NMR spectrum (100 MHz) of compound **1** in $CDCl_3$

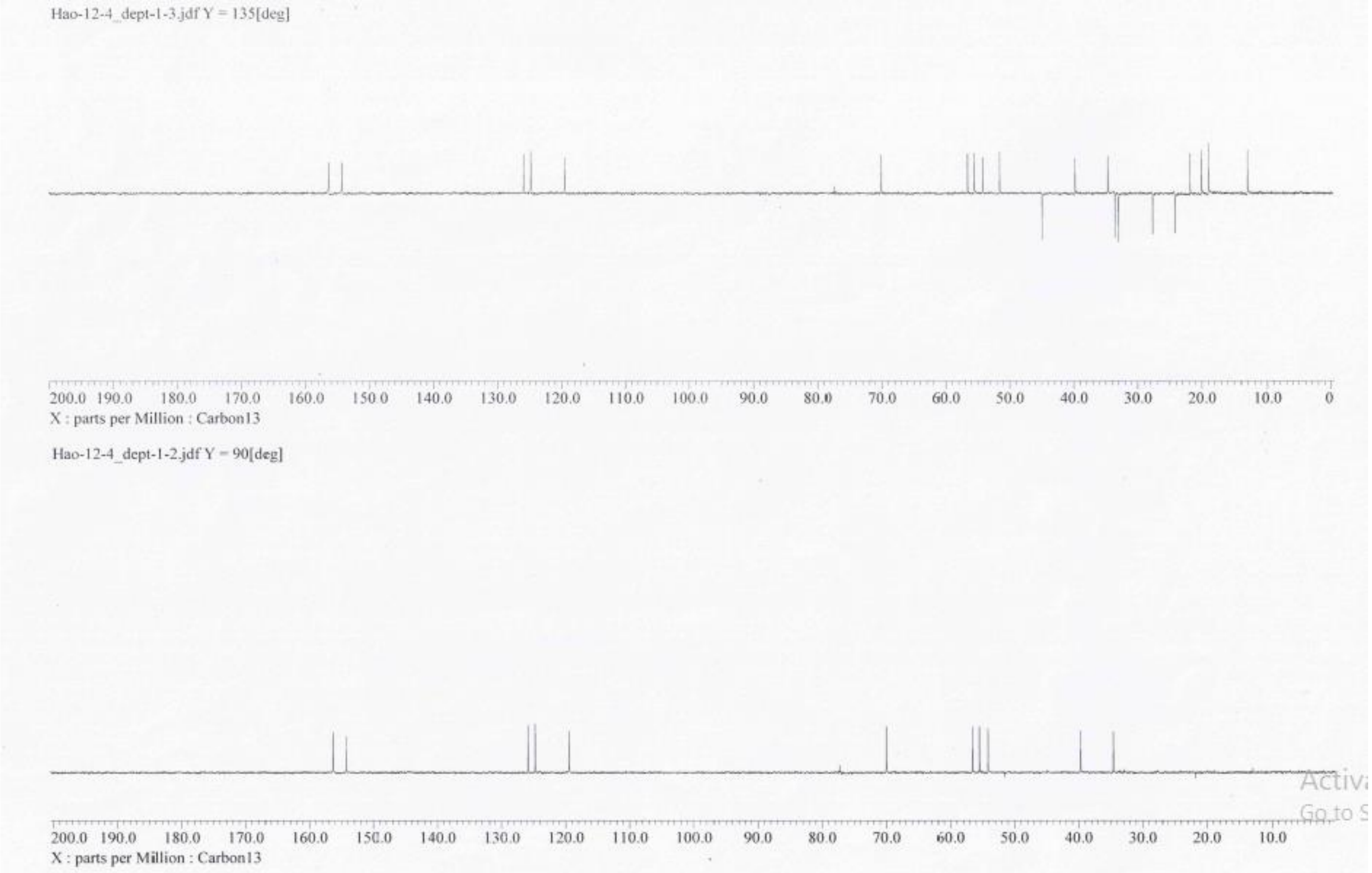

S6. DEPT spectrum (100 MHz) of compound **1** in $CDCl_3$

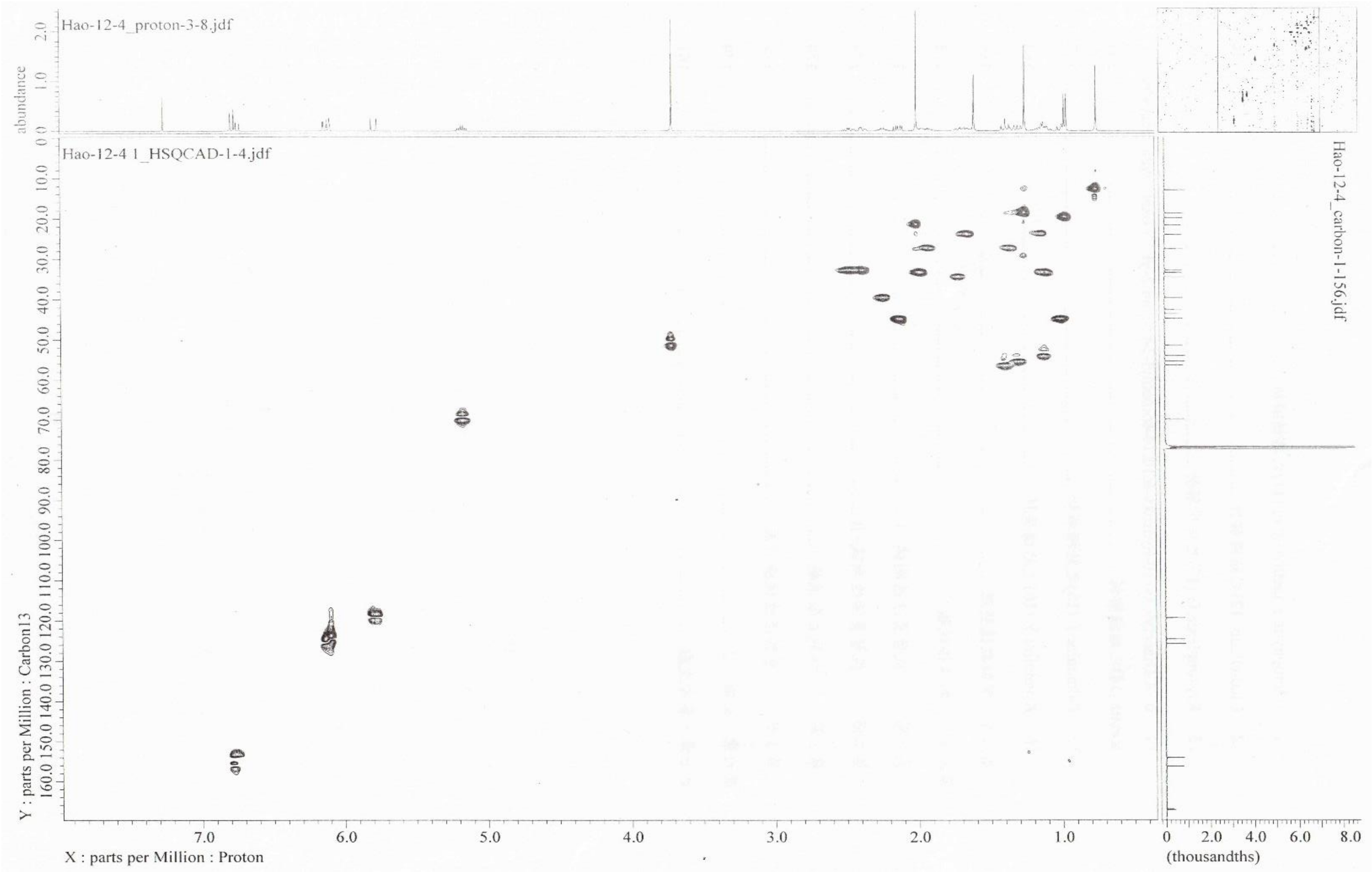

S7. HSQC spectrum of compound **1** in $CDCl_3$

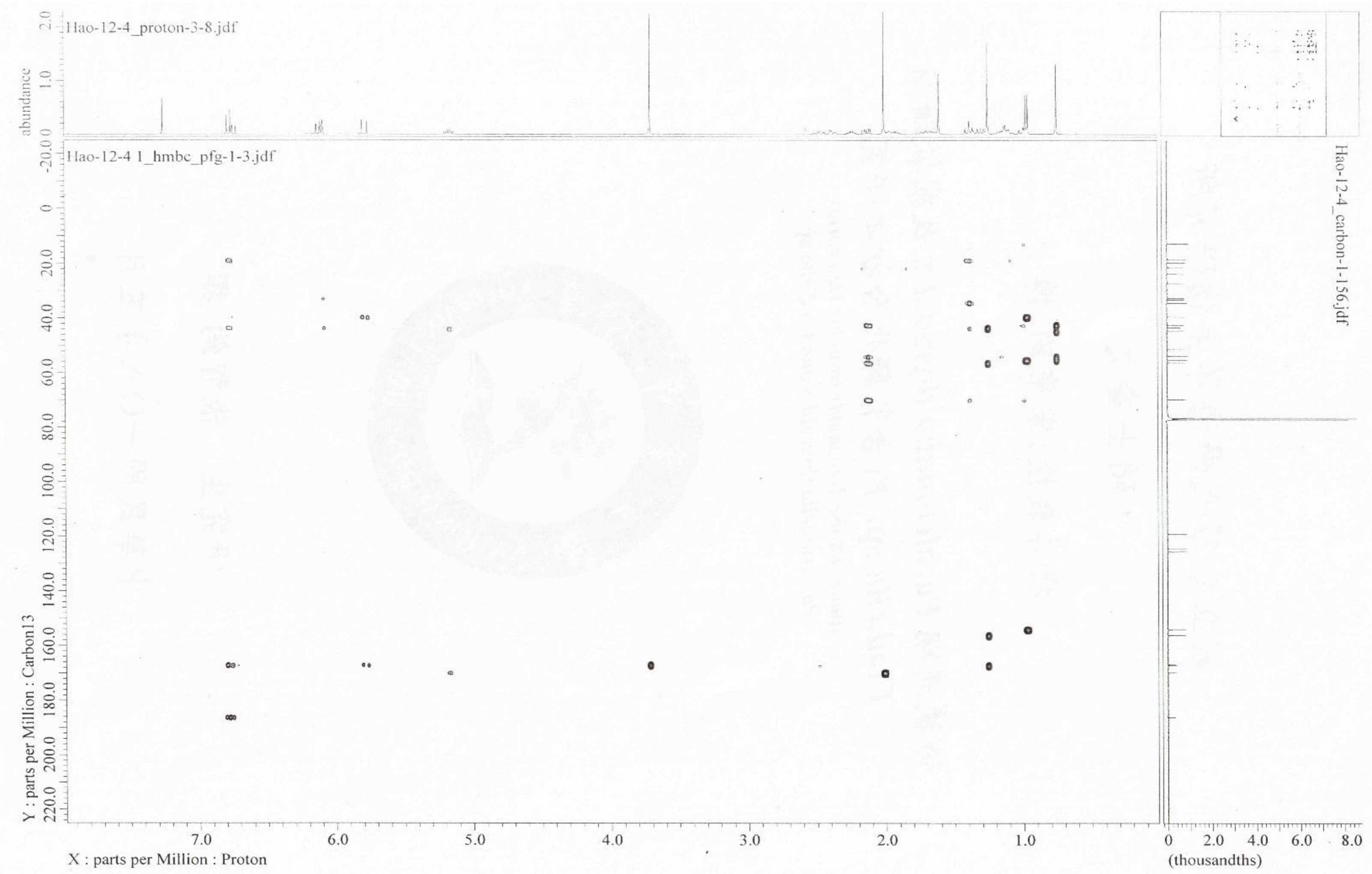

S8. HMBC spectrum of compound **1** in $CDCl_3$

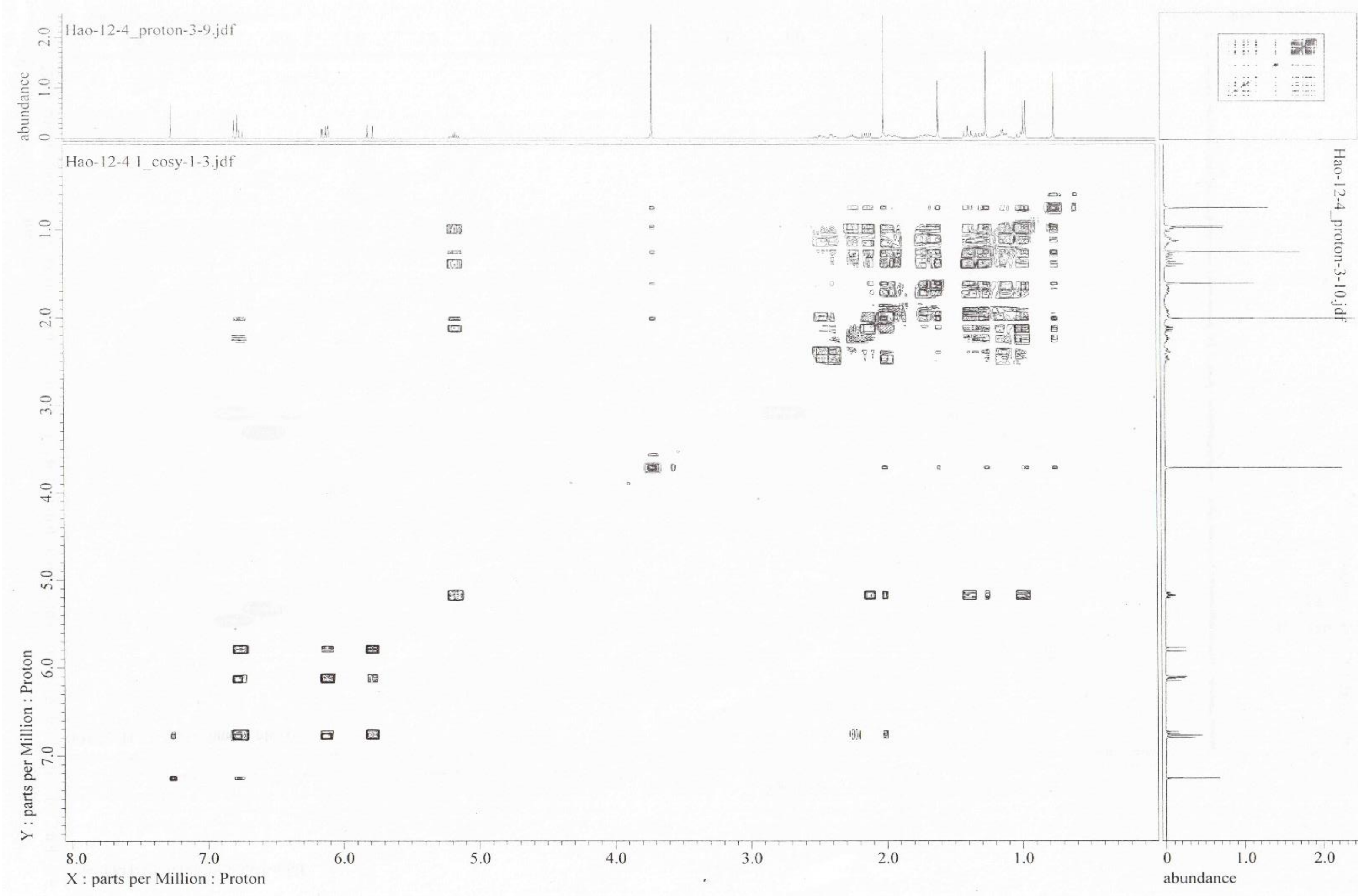

S9. ^{1}H-^{1}H COSY spectrum of compound **1** in $CDCl_3$

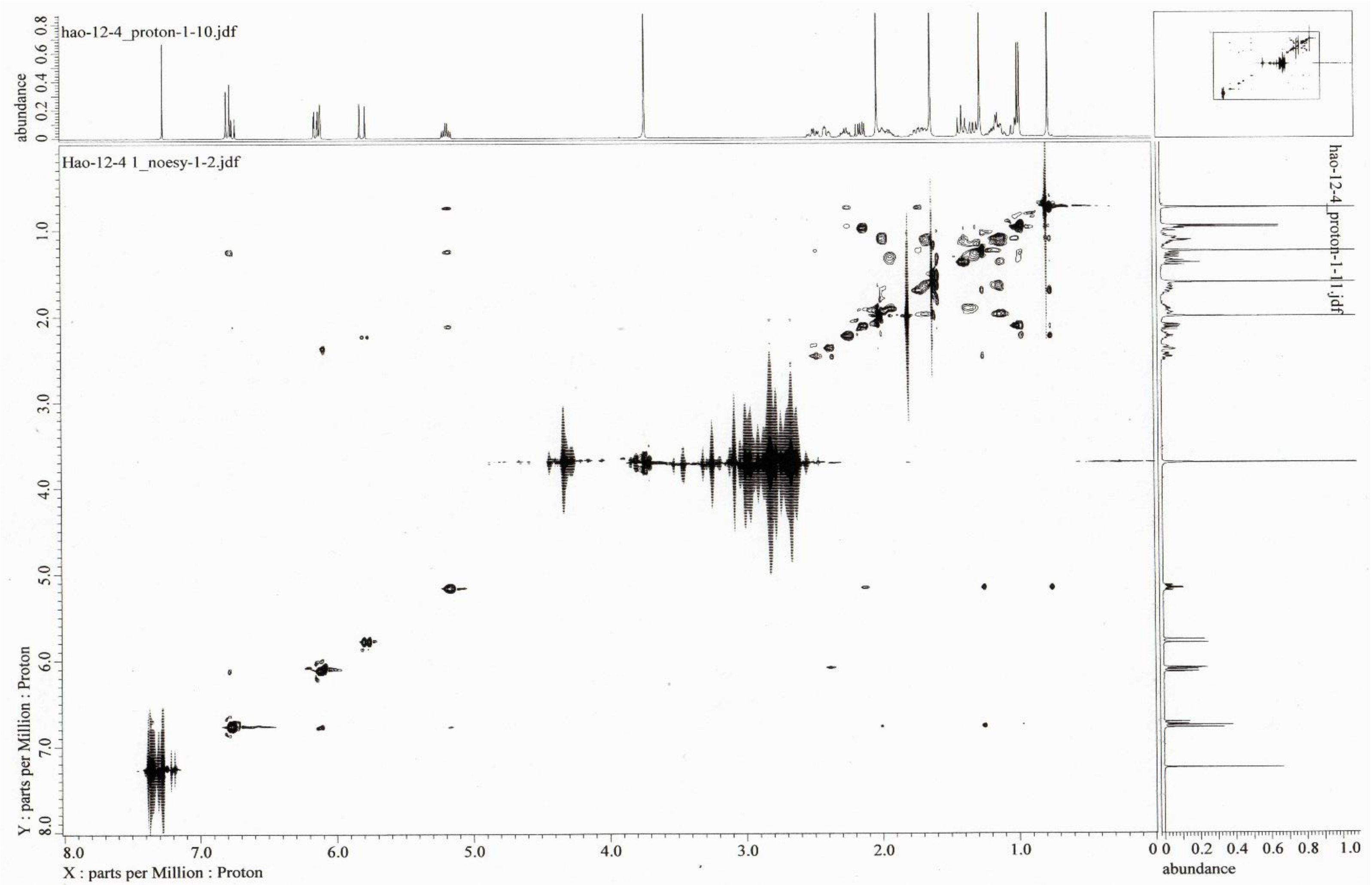

S10. NOESY spectrum of compound **1** in $CDCl_3$

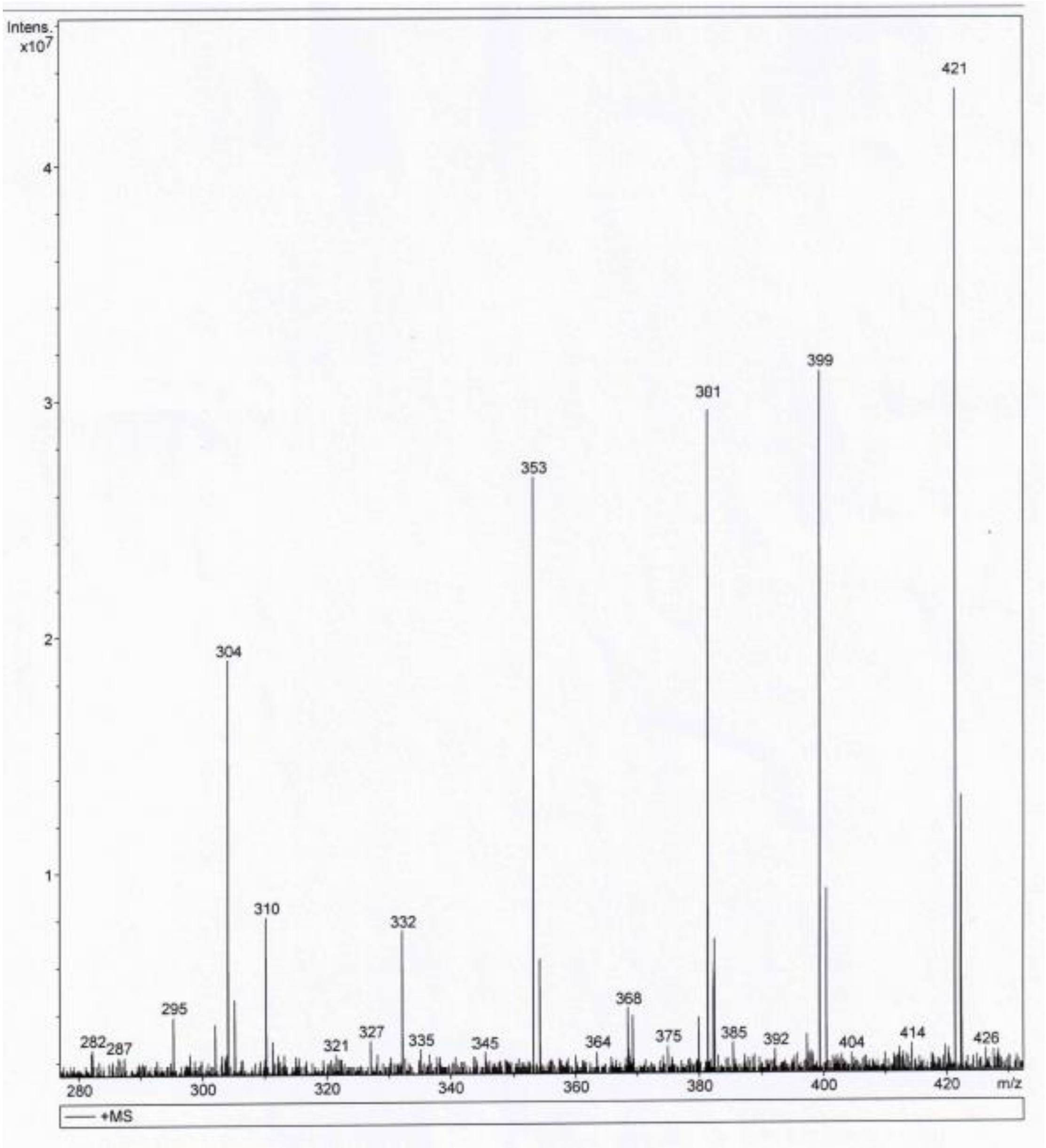

S11. ESIMS spectrum of compound **2**

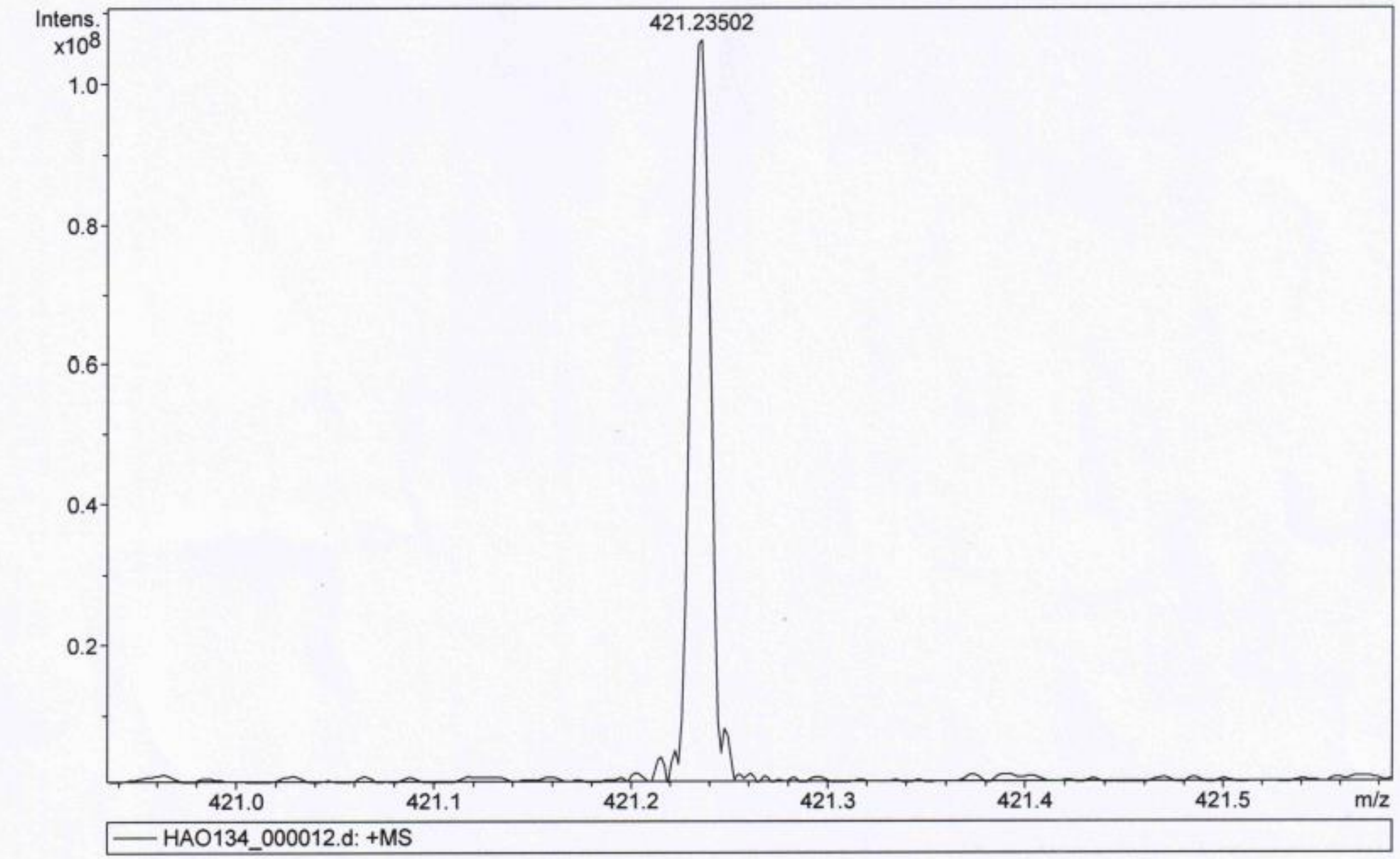

Meas. m/z	#	Formula	Score	m/z	err [mDa]	err [ppm]	mSigma	rdb	e⁻ Conf	N-Rule
421.23502	1	C 25 H 34 Na O 4	100.00	421.23493	-0.09	-0.21	16.7	8.5	even	ok

S12. HRESIMS spectrum of compound **2**

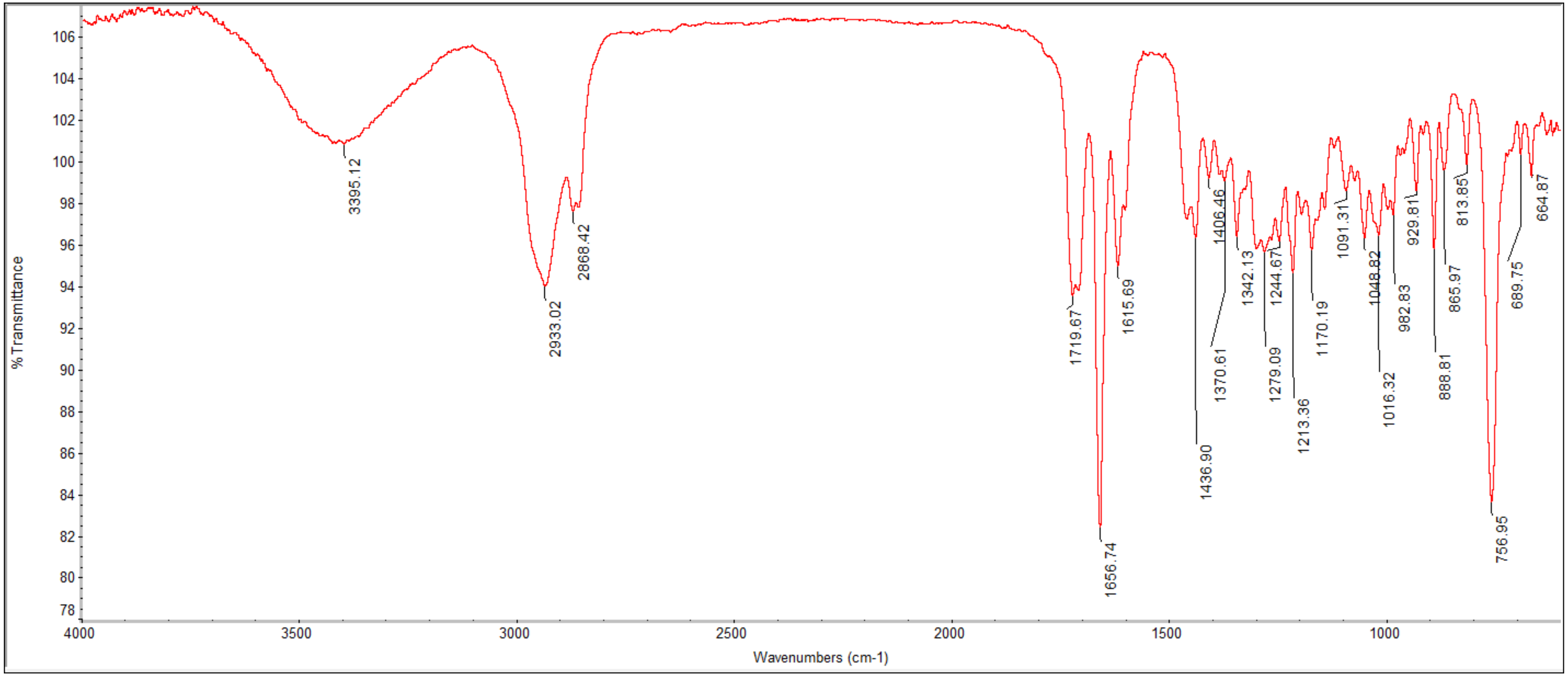

S13. IR spectrum of compound **2**

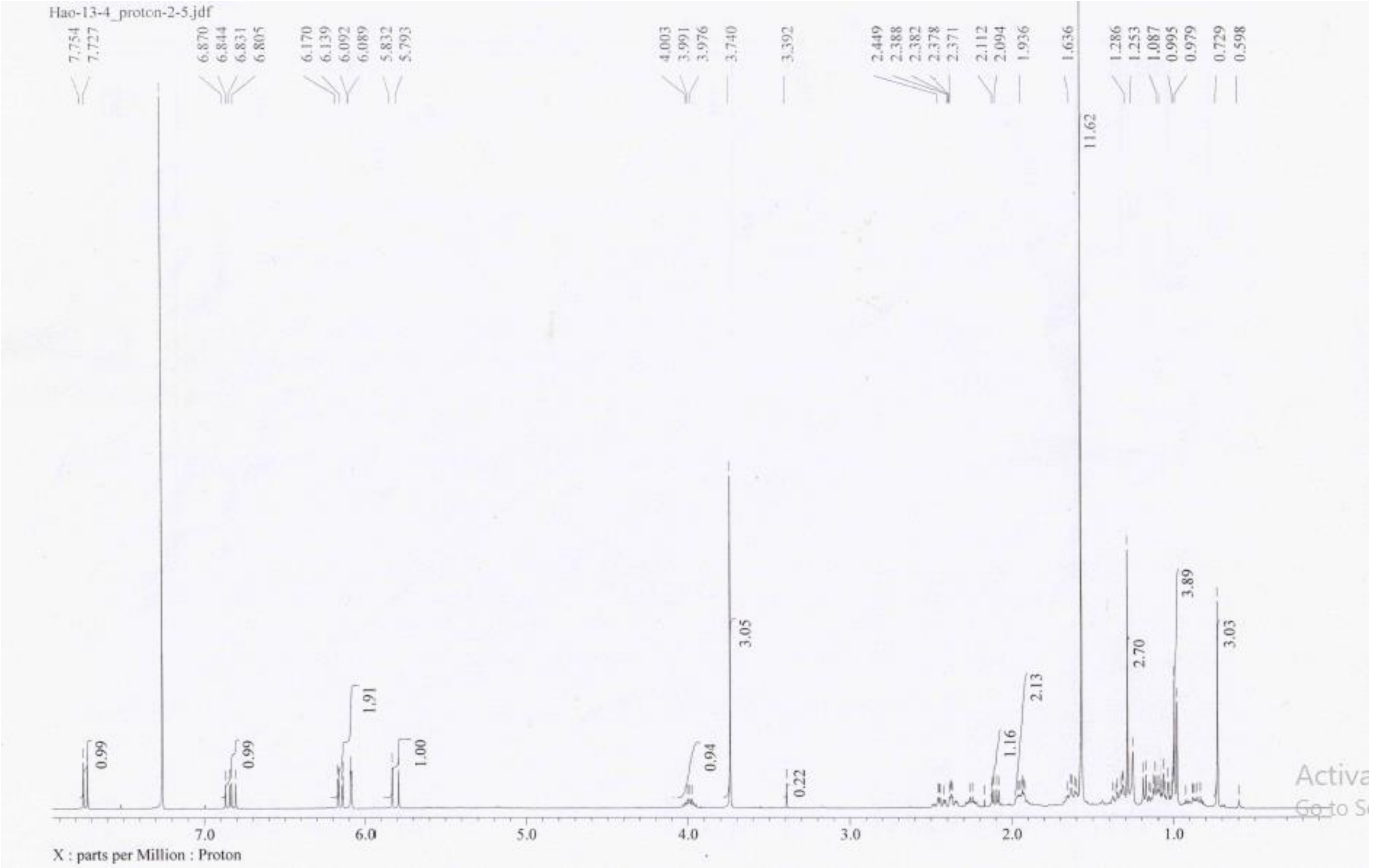

S14. ^{1}H NMR spectrum (400 MHz) of compound **2** in $CDCl_3$

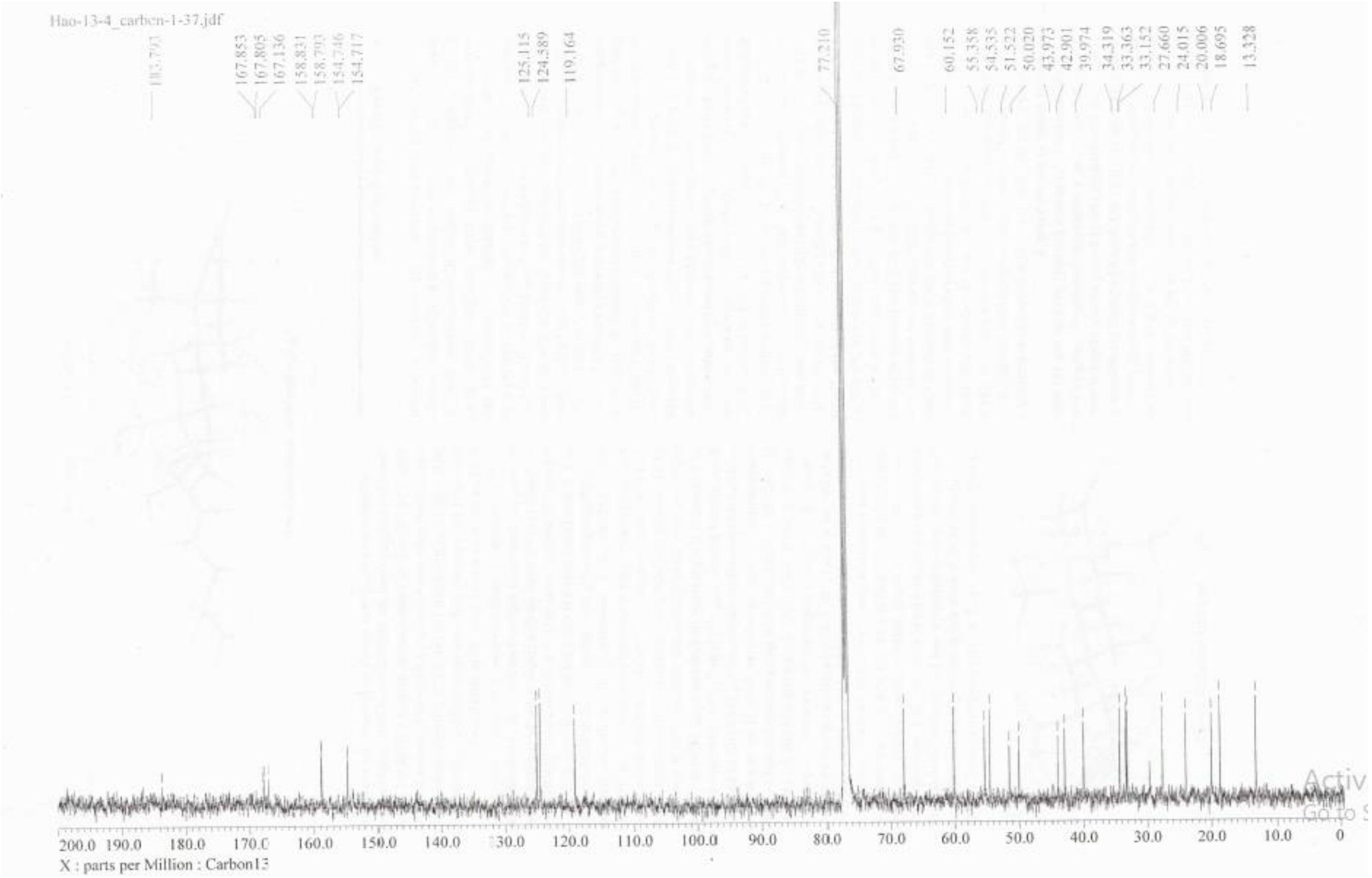

S15. ^{13}C NMR spectrum (100 MHz) of compound **2** in $CDCl_3$

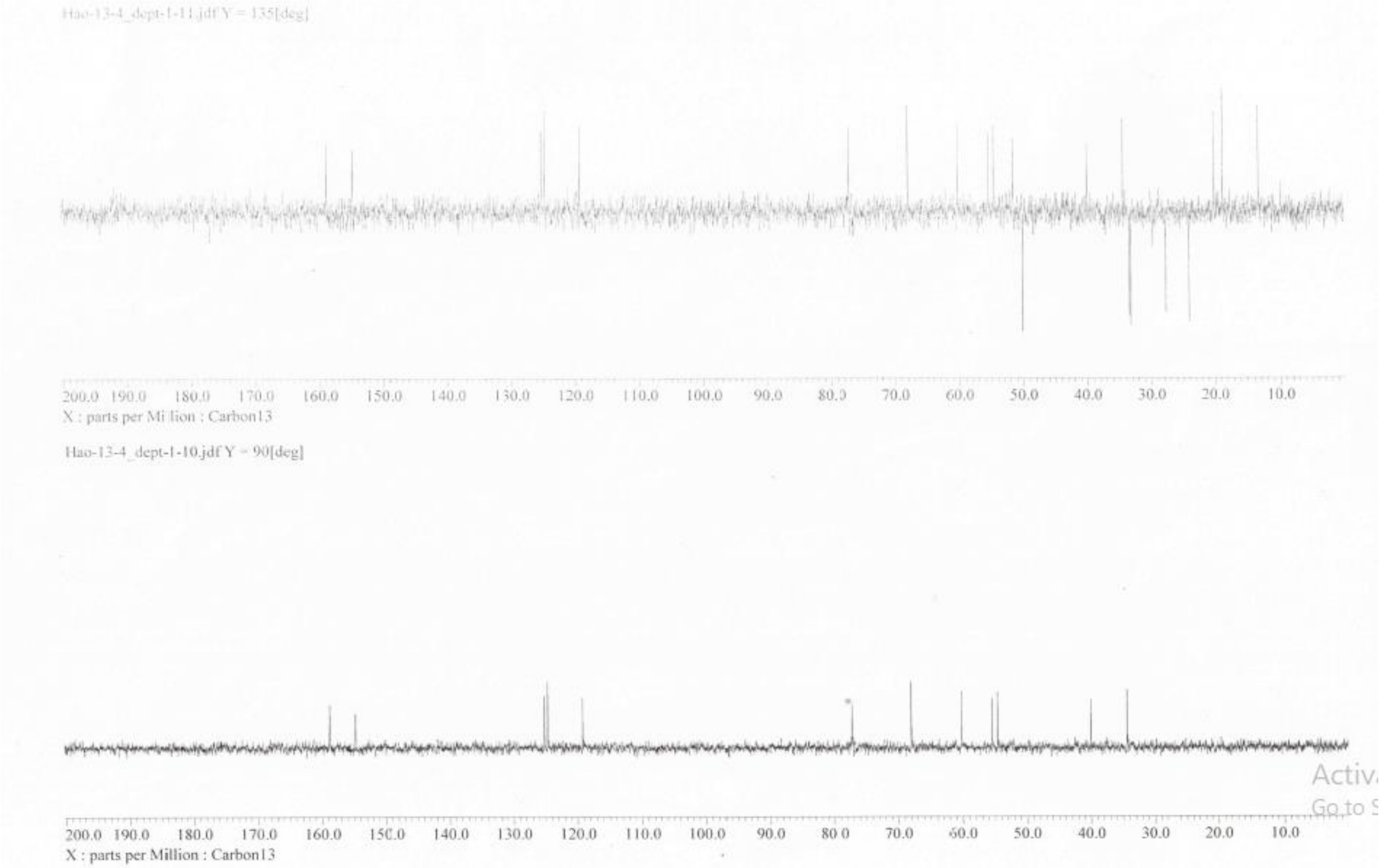

S16. DEPT spectrum (100 MHz) of compound **2** in $CDCl_3$

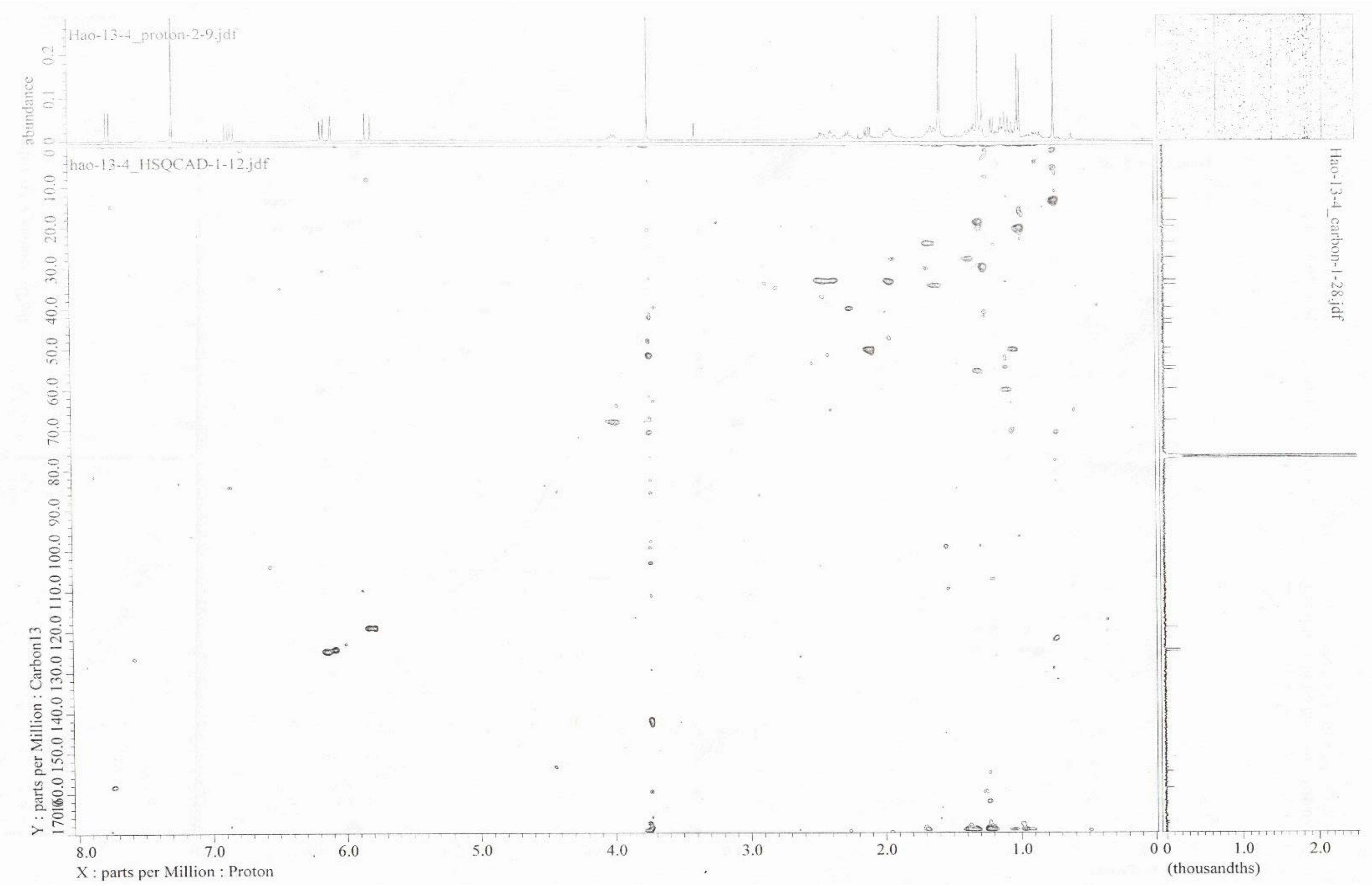

S17. HSQC spectrum of compound **2** in $CDCl_3$

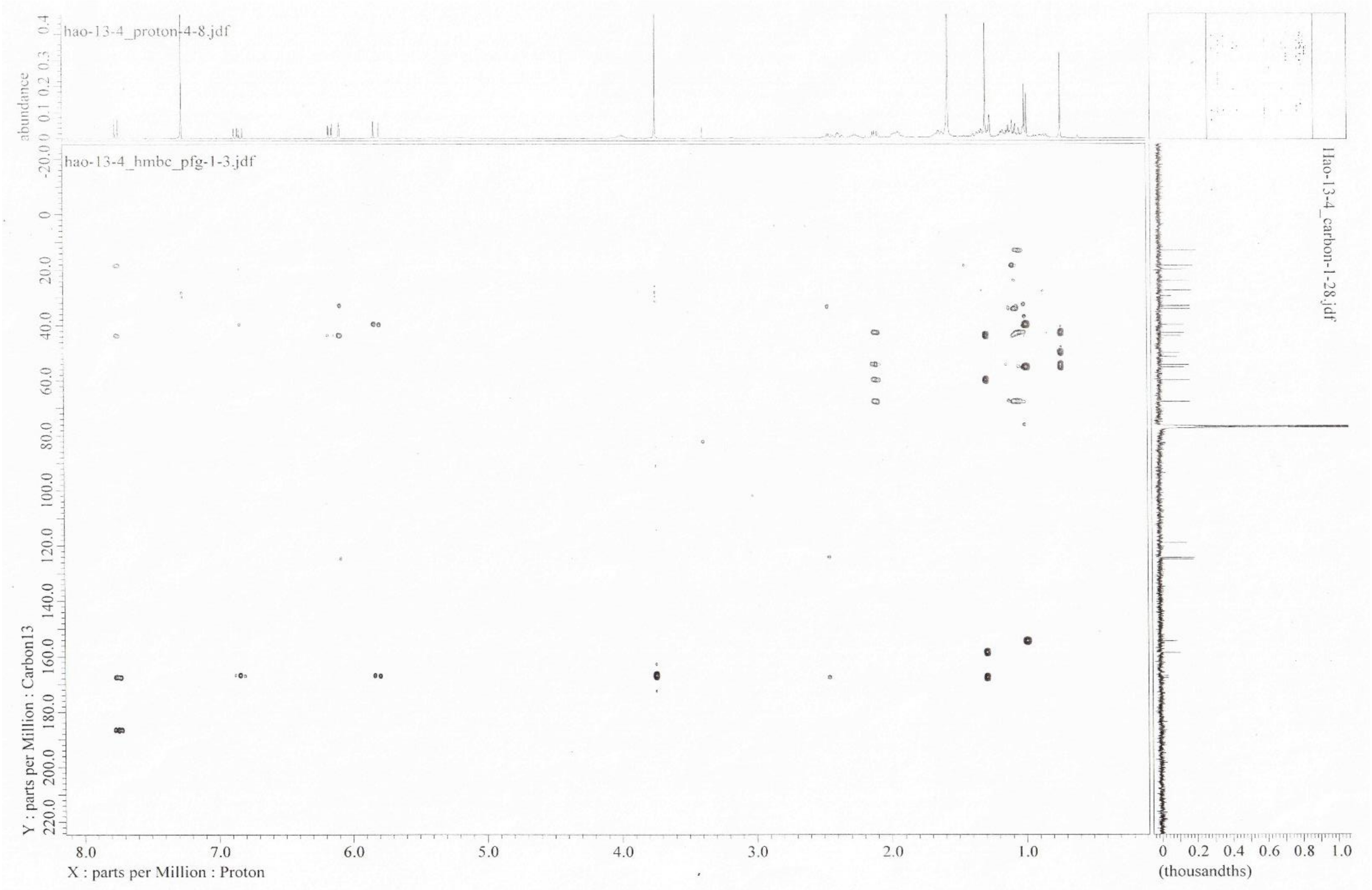

S18. HMBC spectrum of compound **2** in $CDCl_3$

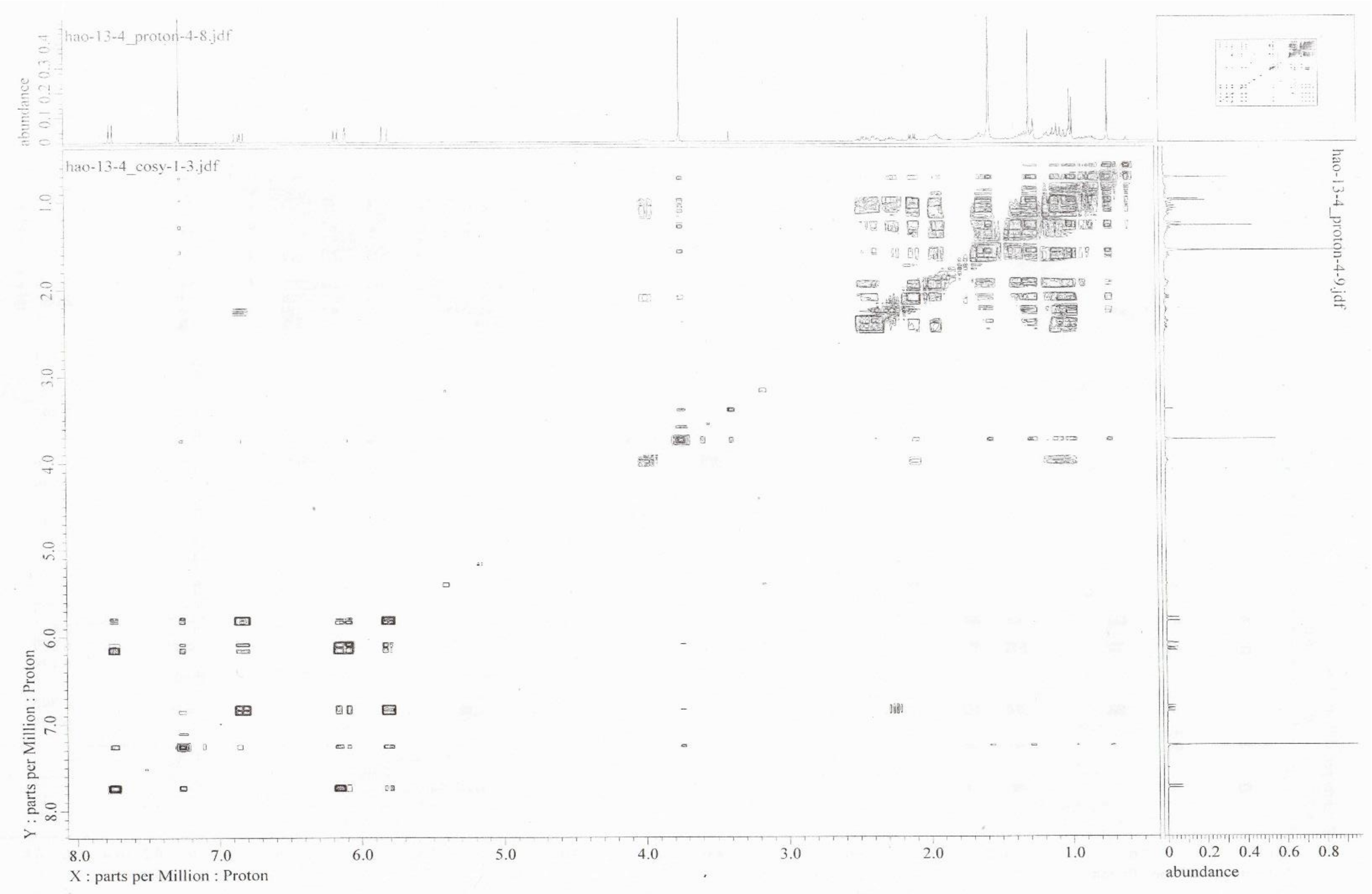

S19. ^{1}H-^{1}H COSY spectrum of compound **2** in $CDCl_3$

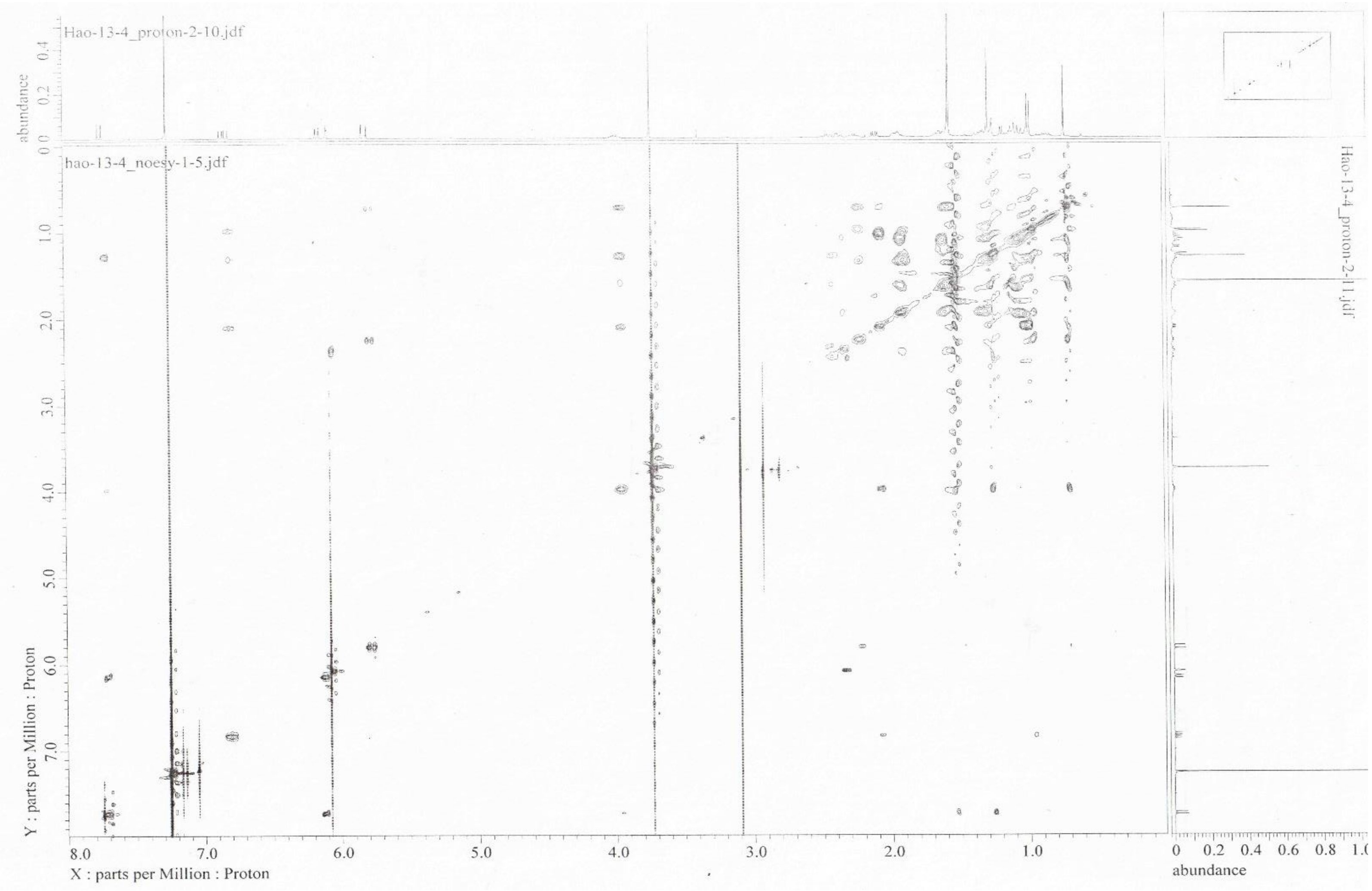

S20. NOESY spectrum of compound **2** in $CDCl_3$

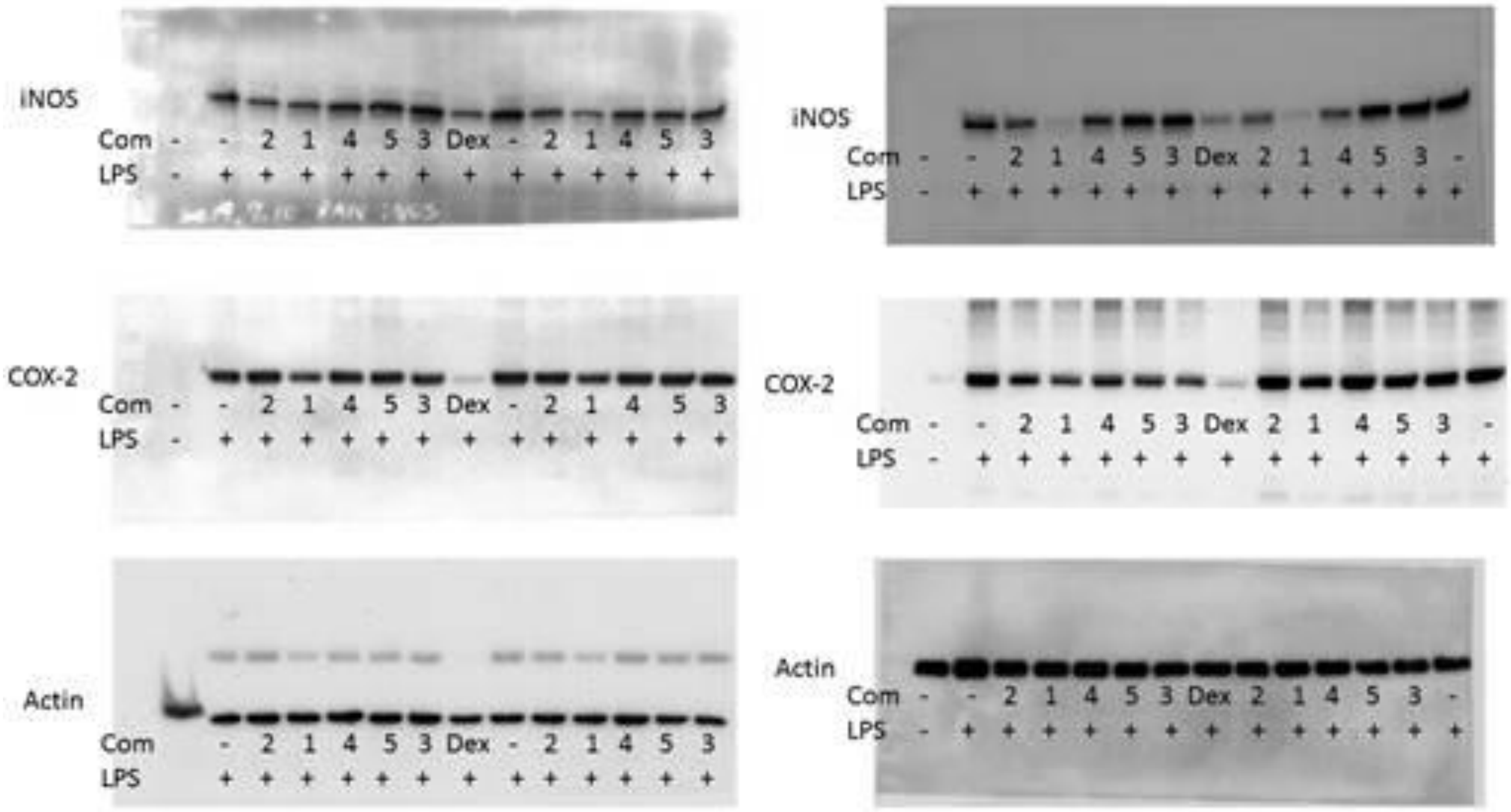

S21. The raw Western blotting data (pictures) of steroids **1**-**5**
